# Targeted Accumulation of Reactive Acrolein via Nanoparticle-embedded Reverse Temperature Hydrogel for Bladder Cancer Treatment

**DOI:** 10.7150/thno.119307

**Published:** 2026-01-01

**Authors:** Yingying Xu, Qinglong Du, Zhongwei Zhao, Shuai Fu, Aijing Zhang, Jianguo Zheng, Chengyang Zhao, Yuxiang Meng, Hanru Liu, Zongyao Lv, Xin Qin, Huimin Geng, Nengwang Yu

**Affiliations:** 1 Department of Urology, Qilu Hospital of Shandong University, Jinan 250012, China.; 2 Cheeloo College of Medicine, Shandong University, Jinan 250012, China.; 3 Department of Medical Oncology, Shandong Cancer Hospital and Institute, Shandong First Medical University and Shandong Academy of Medical Sciences, Jinan 250012, China.; 4 Department of Neurosurgery, Qilu Hospital of Shandong University, Institute of Brain and Brain-Inspired Science, Shandong University, Jinan 250012, China.

**Keywords:** bladder cancer, acrolein, polyamine, nanoparticle, hydrogel

## Abstract

**Rationale:** Bladder cancer (BC) frequently recurs after standard surgical resection and is often associated with poor survival outcomes. Local treatment via intravesical instillation of drug *in situ* is a promising strategy, but its therapeutic efficacy is limited by insufficient retention time and urinary tract obstruction.

**Methods:** We demonstrate that BC exhibits pronounced suppression of acetylpolyamine oxidase (PAOX) compared to normal tissues, as evidenced by multi-platform analysis of patient-derived clinical specimens and GEO database. The PAOX deficiency drives pathological polyamine accumulation in BC cells, uncovering a novel metabolic target with therapeutic implications. Thus, we engineered a novel intravesical instillation system that combines PAOX/targeting peptide-conjugated nanoparticles, synergistically boosting reactive acrolein synthesis while blocking detoxification to induce lethal carbonyl stress, and reverse temperature hydrogels, ensuring liquid dispersion for full bladder coverage during instillation, followed by *in situ* gelation to prolong nanoparticle retention.

**Results:** *In vitro* and *in vivo* results showed that this nanoparticle-embedded hydrogel disrupt redox homeostasis by amplifying lethal carbonyl stress via mitochondrial dysfunction, lipid peroxidation and DNA damage. Remarkably, acrolein binding to GAPDH promotes its nuclear translocation and upregulation of the tumor suppressor protein P53, facilitating apoptosis.

**Conclusion:** This innovative strategy enhances reactive acrolein accumulation by targeting tumor-specific metabolic vulnerabilities, inducing multifaceted cell death for the BC treatment.

## Introduction

Bladder cancer (BC) is among the most prevalent malignancies of the urinary tract, accounting for approximately 550,000 new cases and over 200,000 deaths annually worldwide 1,2. Although the majority of BC cases are diagnosed at the non-muscle-invasive stage, approximately 70% of these progress to muscle-invasive bladder cancer (MIBC), with a median survival of 1~5 years 3,4. Current therapeutic for BC typically involves transurethral resection followed by postoperative intravesical chemotherapy. Traditional chemotherapy drugs, such as Bacillus Calmette-Guérin (BCG) and mitomycin C, were dissolved in appropriate solvents, and then administered for intravesical instillation 5. The drug retention times is less than 2 h due to the rapid urinary clearance, which significantly reduces drug residence time within tumor tissues and limits the therapeutic efficacy 6. Moreover, the high concentrations of chemotherapeutic agents required to achieve clinical benefit are often associated with severe adverse effects, including infection, inflammation, and hematuria, contributing to recurrence rates as high as 50-70% 7,8. Therefore, it is crucial to explore novel therapeutic strategies that enhance drug retention time and efficacy while minimizing adverse effects.

Elevated levels of urinary spermine have been reported in BC patients, particularly those with MIBC. Increased urinary polyamines (PAs) are consistently associated with tumor tissues and have been identified as potential biomarkers for poor prognosis 9,10. Recent studies have further demonstrated that PAs supplementation can sensitize cancer cells to radiotherapy or chemotherapy by triggering ferroptosis, highlighting PAs metabolism as a targetable therapeutic vulnerability in cancer 11. Interestingly, intracellular PAs can be oxidized by plasma amine oxidase (PAO), producing substantial quantities of hydrogen peroxide (H_2_O_2_) and acrolein (ACR), thereby initiating and sustaining tissue damage 10. ACR, one of the most reactive aldehydes, exhibits pronounced electrophilicity and forms proteins and DNA adducts via Michael addition reaction, inducing carbonyl stress that potentiate oxidative stress-induced tumor cytotoxicity 12. This indicates that ACR can disrupt the redox balance and exhibits significant anti-cancer potential. However, tumor cells often exhibit elevated glutathione (GSH) levels, a major antioxidant, which acts as the primary defense mechanism against ACR. High GSH concentrations enhance the detoxification of reactive oxygen species, providing cancer cells with a survival advantage 13,14. Therefore, based on the upregulation of PAs in BC cells, boosting ACR accumulation and inducing lethal carbonyl stress via oxidation-mediated ACR production and GSH depletion is a promising therapeutic strategy.

Direct bladder instillation of drug solutions allows efficient diffusion of therapeutic agents across the bladder wall. However, this approach is limited by rapid drug washout due to urinary voiding, compromising therapeutic efficacy. Owing to the bladder's unique luminal architecture, nanodrug delivery systems have shown superior targeting compared to conventional chemotherapeutics 4,15. Nevertheless, it is also accompanied by several challenges, including susceptibility to urine-mediated drug loss and instability over prolonged exposure 16. Alternatively, hydrogels offer the advantage of controlled release and prolonged bladder retention 17,18. However, their intricate network structure can impede drug mobility, reducing bioavailability. To overcome these limitations, a dual-phase sustained-release system integrating both solution and gel phases presents an optimal strategy for intravesical therapy 19-22. This system capitalizes on the fluidity of solutions for uniform drug distribution while harnessing the encapsulating properties of hydrogels to sustain drug release and mitigate urine washout, ultimately enhancing therapeutic outcomes and minimizing drug loss.

**Scheme 1:**
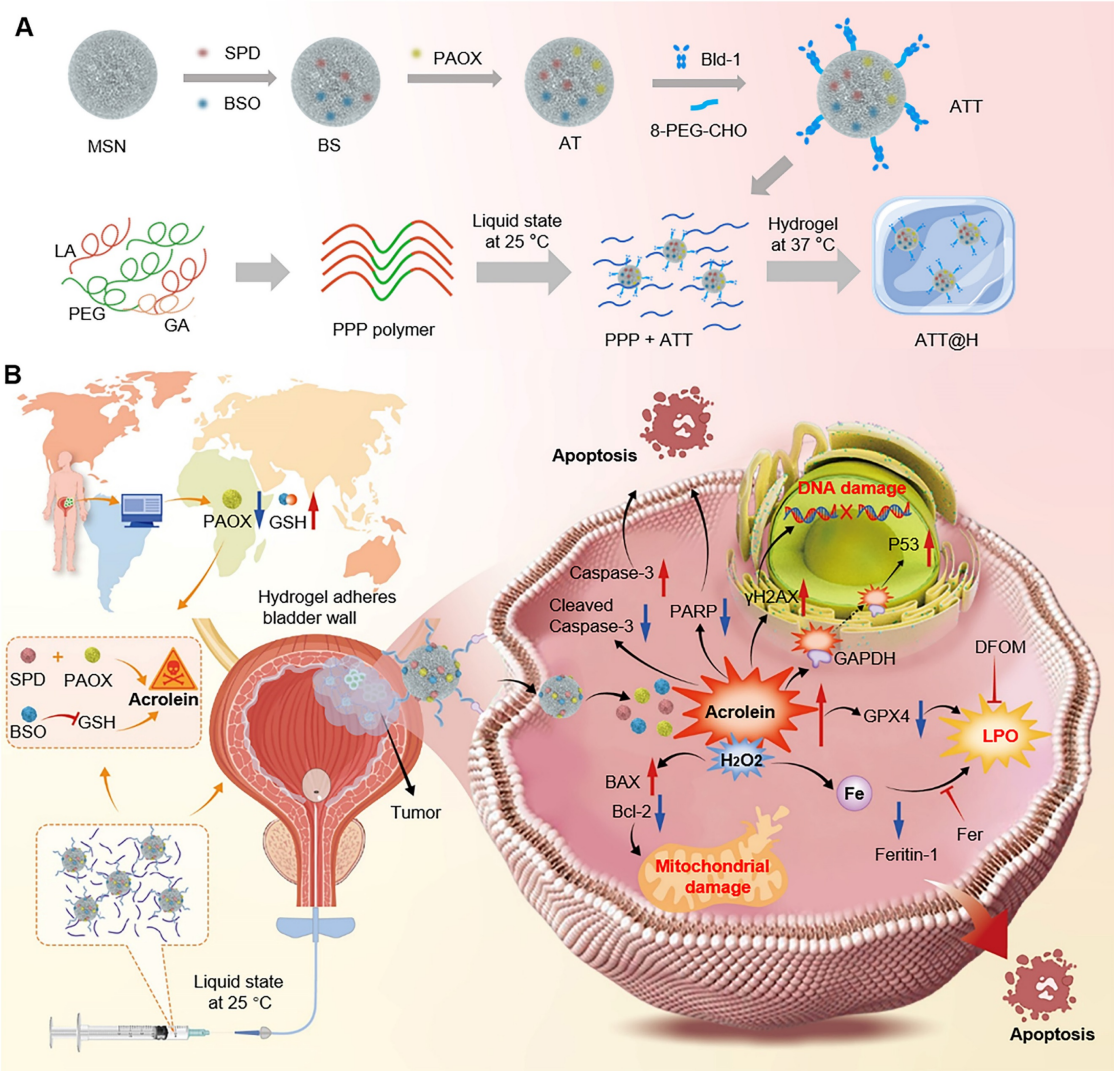
**(A)** Preparation of the nanoparticle-hydrogel system. **(B)** Schematic illustration of ACR-triggered nanoparticle-embedded reverse temperature hydrogel preparation and potential mechanisms for BC treatment.

In this study, we observed low acetylpolyamine oxidase (PAOX) expression in BC tissues than that of in normal tissues via clinical samples and GEO database, which derived PAs accumulation and limited ACR generation. Exploiting the BC microenvironment characterized by elevated PA levels and downregulation of PAOX as potential therapeutic target, we develop a nanoparticle-hydrogel composite system to improve intravesical drug retention and cascade boost the deposit of intracellular ACR for effective treatment. First, ACR-triggered nanoparticles (NPs) are prepared by co-loading spermidine (SPD) and DL-Buthionine-(S,R)-sulfoximine (BSO), then functionalized with PAOX and a bladder-targeting peptide. The multi-functional NPs enhanced ACR production from SPD and simultaneously inhibited GSH-mediated detoxification, inducing potent carbonyl stress that intensified oxidative damage to disrupt redox homeostasis *in vivo* and triggering multifaceted tumor cell death. In parallel, a temperature-responsive poly(lactic acid-co-glycolic acid)-poly(ethylene glycol)-poly(lactic acid-co-glycolic acid) copolymer (PLGA-PEG-PLGA, PPP) was synthesized, remaining in sol state below 37 °C and transforming into a gel at body temperature. Upon intravesical instillation, the liquid state ensures widespread NPs dispersion throughout the bladder, targeting tumor cells effectively. Subsequent gelation sustains drug release and extends the duration of action (Scheme 1). This NPs-hydrogel instillation system enables accumulation of reactive ACR, offering a potential therapeutic target for BC.

## Results and Discussion

### Low expression of PAOX in bladder cancer

**Figure 1:**
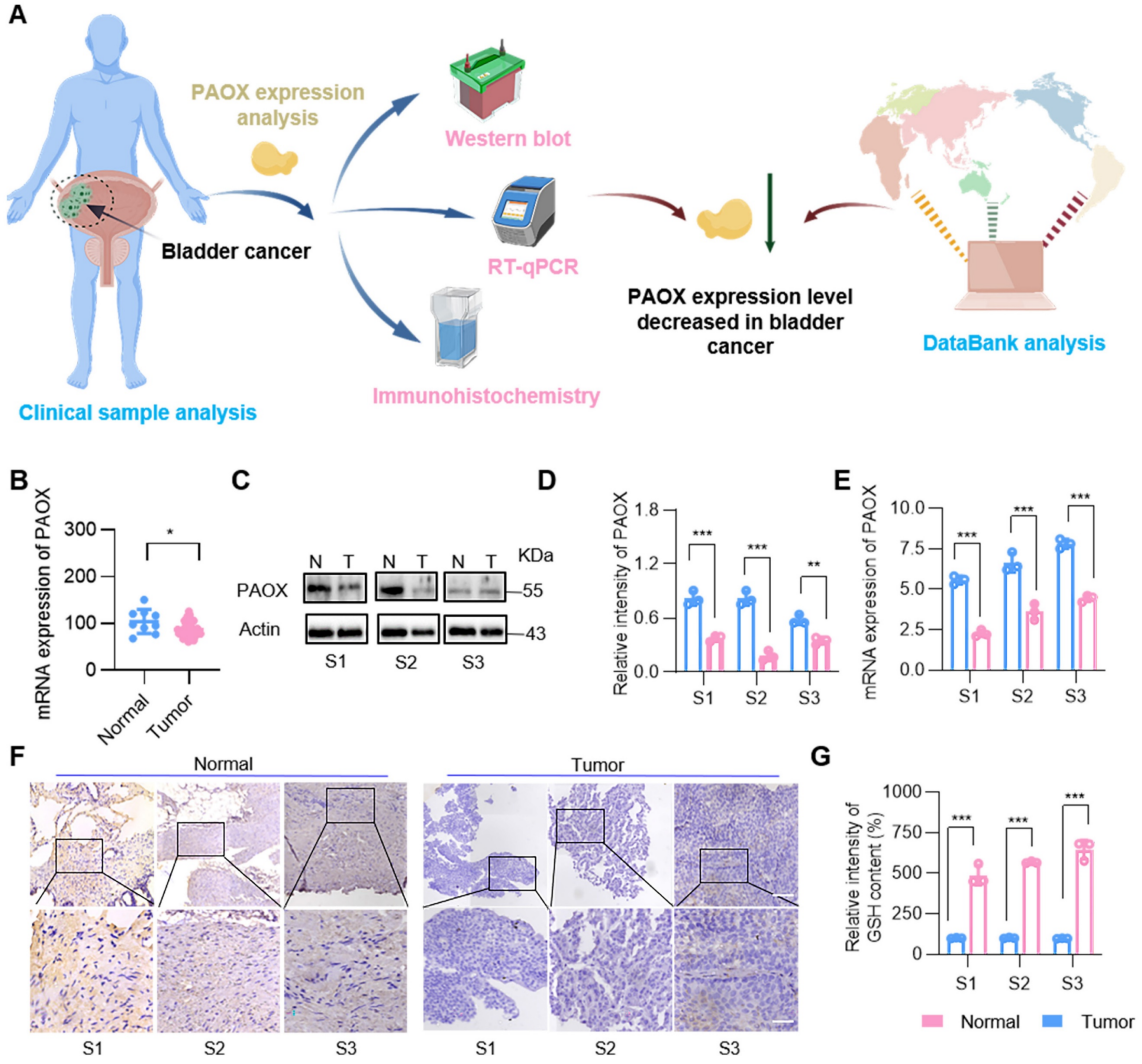
**PAOX expression in human BC tissues and adjacent tissues. (A)** Diagram showing the experimental procedure for PAOX expression analysis. **(B)** Quantification of PAOX mRNA levels in human BC tissues (n = 41) and normal tissues (n = 9) from the GEO database (GSE3167). **(C, D)** Western blot analysis of PAOX expression levels in clinical human BC samples. N, normal tissues; T, tumor tissues. **(E)** mRNA levels of PAOX in clinical human BC samples. **(F)** Representative IHC images of PAOX expression in clinical human BC samples. Scale bars in low magnification images represent 500 μm, and in high magnification images represent 100 μm. **(G)** GSH levels in clinical human BC samples. Data are presented as mean ± s.d. *p < 0.01; **p < 0.01; ***p < 0.001 by two-way ANOVA test. The schematic diagram was created with BioGDP.com 30.

Although the toxic substances are produced during the PAs oxidation to effectively kill tumor cells, the cytotoxicity is not pronounced due to the low expression of amine oxidase in cancer cells, such as prostate cancer cells 23,24. To confirm the reduced expression of PAOX in clinical bladder tumors, we evaluated its levels using GEO database analysis, Western blot, RT-qPCR and immunohistochemistry (IHC) (Figure 1A). Database results indicated that PAOX expression in BC tissues was slightly lower than in normal tissues (Figure 1B). Analysis of 3 clinical bladder cancer specimens revealed reduced PAOX mRNA and protein levels in tumor tissues compared to adjacent normal tissues. (Figure 1C-E). IHC further confirmed cytoplasmic localization of PAOX, with significantly diminished expression in cancerous tissues (Figure 1F). Polyamine homeostasis varies between normal and tumor cells 25, and we next assessed the expression of key enzymes involved in PA metabolism. In the BC database, mRNA levels of enzymes such as spermidine/spermine N^1^-acetyltransferase 1 (SAT1) and spermine oxidase (SMOX) were down-regulated relative to normal tissues, while ornithine synthetase (ODC1) was upregulated. Additionally, ATP13A2, a PAs transporter 26,27, showed elevated expression in BC (Figure S1), suggesting increased intracellular PAs availability. Further analysis demonstrated significantly higher GSH levels in tumor tissues (Figure 1G). Collectively, these findings indicate that although PAs are abundant in BC cells, their oxidation is constrained by insufficient PAOX expression and robust GSH-mediated antioxidant defenses—factors that undermine the tumoricidal potential of PA oxidation products.

To evaluate the cytotoxic potential of PA oxidation products, a pCDNA3.1-PAOX plasmid was transfected into BC cells, and cell proliferation was assessed using the MTT assay. Following transfection, PAOX expression was observed for 48 h, and mRNA levels of other PA metabolism-related enzymes, including SAT1 and SMOX, were also upregulated. Cell viability significantly decreased post-transfection by more than 10%, and further incubation with SPD with a concentration of 200 μM led to an even greater reduction by 20% in cell viability (Figure S2). Among the PA oxidation products, ACR is the most cytotoxic, exerting its effects at micromolar concentrations. However, its toxicity is attenuated by GSH-mediated detoxification 28,29. To inhibit ACR clearance, we co-treated cells with the GSH synthesis inhibitor BSO and evaluated cell proliferation. The results showed that in the BSO-treated group (50 µM), cell viability declined by more than 95%. Notably, while BSO and SPD exhibit cytotoxicity only at millimolar concentrations after prolonged exposure, ACR demonstrated potent cytotoxicity at nanomolar concentrations, with an IC value of 100 nM to exert its effects. In combination treatment, the BSO dose was relatively low and only mildly cytotoxic on its own (Figure S3).

### Characterization of the co-delivery drug system

**Figure 2:**
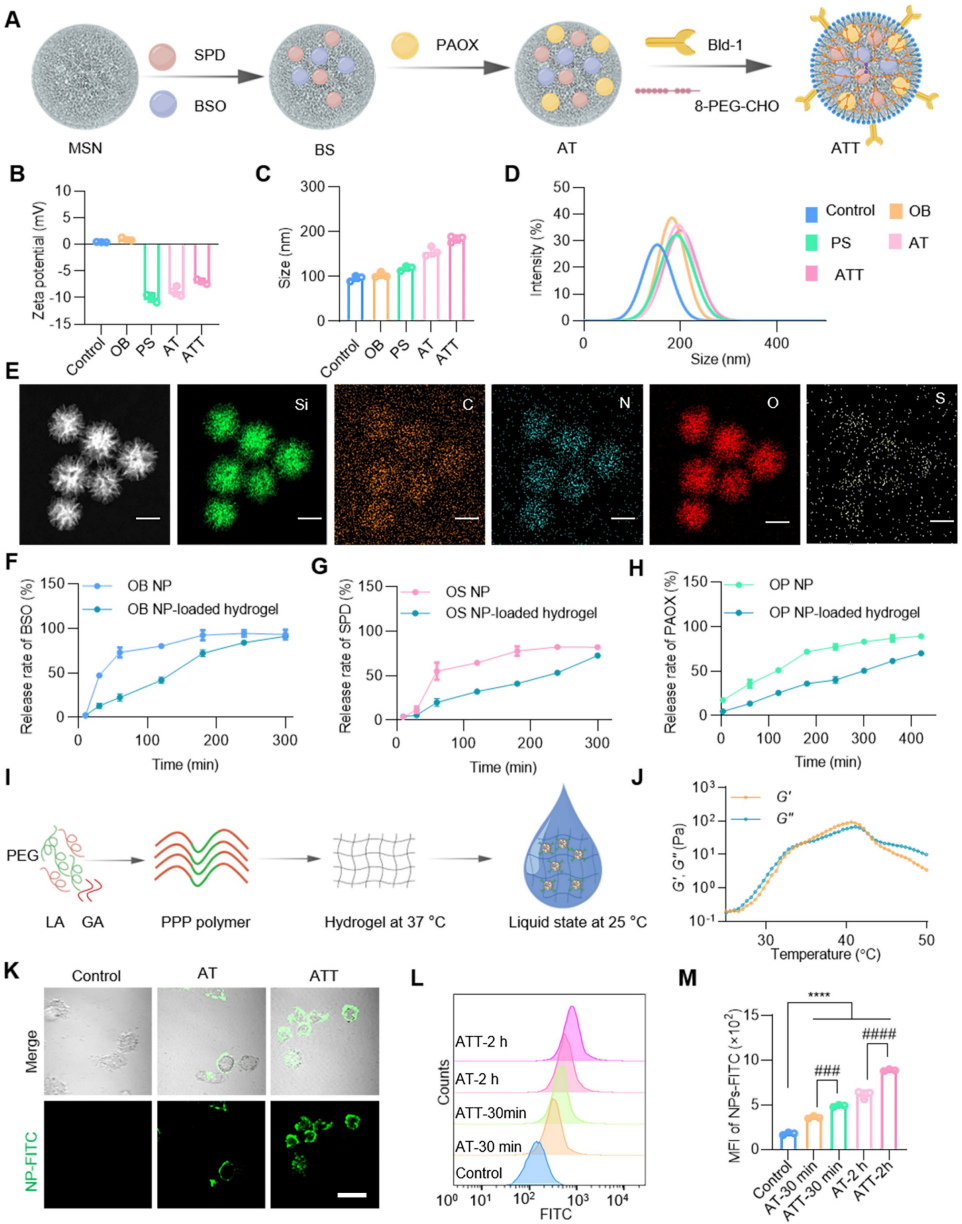
**Synthesis procedure and characterization of NP-hydrogel systems. (A)** Schematic illustration showing ATT NP preparation. **(B)** Zeta potential of each nanoparticle (n = 3). **(C)** Size distributions of each nanoparticle (n = 3). **(D)** Dynamic light scattering analysis of NPs. **(E)** EDS mapping of ATT NPs, scale bar, 100 nm. Release rate of BSO **(F)**, SPD **(G)** and PAOX **(H)** from OB, OS and OP NPs and NP-loaded hydrogels in PBS solution at 37°C, respectively. **(I)** Diagram showing the PPP polymer composed of poly(ethylene glycol) (PEG), _D,L_-Lactide (LA) and glycolide (GA) to prepare temperature-responsive hydrogels. **(J)** Temperature sweep of storage moduli (G′) and loss moduli (G″) of PPP hydrogels. **(K)** Cellular uptake of NPs with or without Bld-1 by BC cells MB49. NPs-FITC, green. Scale bar indicates 20 μm. **(L, M)** Flow cytometry analysis and quantification of cellular uptake of NPs. Data are presented as mean ± s.d. n = 3. ###p < 0.001; ####p < 0.0001; ****p < 0.001 by one-way ANOVA test.

Inspired by the effectiveness of the PAOX-SPD-BSO combination, we developed a strategy to trigger ACR *in situ* using PAOX and SPD while simultaneously inhibiting its detoxification via BSO. For application at the individual level, a drug delivery system based on mesoporous silica nanoparticles (MSNs) was constructed (Figure 2A). SPD and BSO were co-encapsulated into MSNs, forming BS NPs, with an average particle size of ~104.22 nm. These NPs were subsequently coated with PAOX to prepare ACR-triggered NPs (defined as AT). To boost targeting efficacy to bladder tumor cells, a targeting peptide—Bld-1 (sequence CSNRDARRC) was grafted onto AT NPs using 8-arm-poly(ethylene glycol)-benzaldehyde (8-PEG-CHO) 31,32, yielding ATT NPs with an average size of 185.66 nm, and zeta potential of -6.97mV. Moreover, we prepared OB NPs that MSNs only loaded BSO, and PS NPs that MSNs encapsulated with PAOX and SPD to validate the comparative assessment (Figure 2B-D). These designs' features support the effective exploitation of tumor-specific metabolic profiles and microenvironments for cancer therapy.

The successful synthesis of ATT was confirmed by energy dispersive spectrometry mapping (Figure 2E). The maximum absorbance peaks of BSO and SPD were detected at 202 nm and 200 nm respectively, with a linear correlation between absorbance and concentration in the ranges of 0.01~0.5 µg and 0.35~0.5 µg, respectively (Figure S4A-B), which were used to determine encapsulation and release efficiencies. The drug loading for SPD and BSO was calculated to be 82% ± 3.23% and 85% ± 5.61%, respectively. Both BSO and SPD were rapidly released within 3 h, with a release rate of ~90%, followed by sustained release over 8 h (Figure 2F-G). PAOX was loaded over 95% and exhibited slower release kinetics, with remaining 20% after 6 h (Figure 2H). To enhance NP perfusion in the bladder, a thermo-responsive triblock copolymer PPP was synthesized via stannous octoate-catalyzed ring-opening polymerization (Figure S4C-D). The copolymer remained in sol form at room temperature and transitioned to a gel at body temperature (Figure 2I-J and Figure S4E). MSNs were uniformly dispersed in the PPP hydrogel, and the embedded formulation retained its thermogelling property (Figure S4F). As depicted in Figure 2F-H, the hydrogel exhibited a capacity to retard the release of BSO, SPD, and PAOX from NPs. These results confirm the successful construction of a stable, dual-phase NP-hydrogel drug delivery system with controlled release capabilities. To further investigate cell-targeting specificity of the NPs, AT and ATT were labelled by fluorescein isothiocyanate (FITC). Compared with AT, the ATT demonstrated rapid cellular uptake within 10 min, with progressively enhanced intracellular fluorescence (Figure 2K, Figure S5). Flow cytometry analysis showed that AT had significantly higher cellular association than non-targeted particles (Figure 2L-M), confirming the targeting efficiency of Bld-1. These results suggest that the successful construction of the system and precise tumor targeting by Bld-1, which will promote the utilization of drugs absorbed by cells.

### Lethal effects of drug delivery system on bladder cancer cells *in vitro*

**Figure 3:**
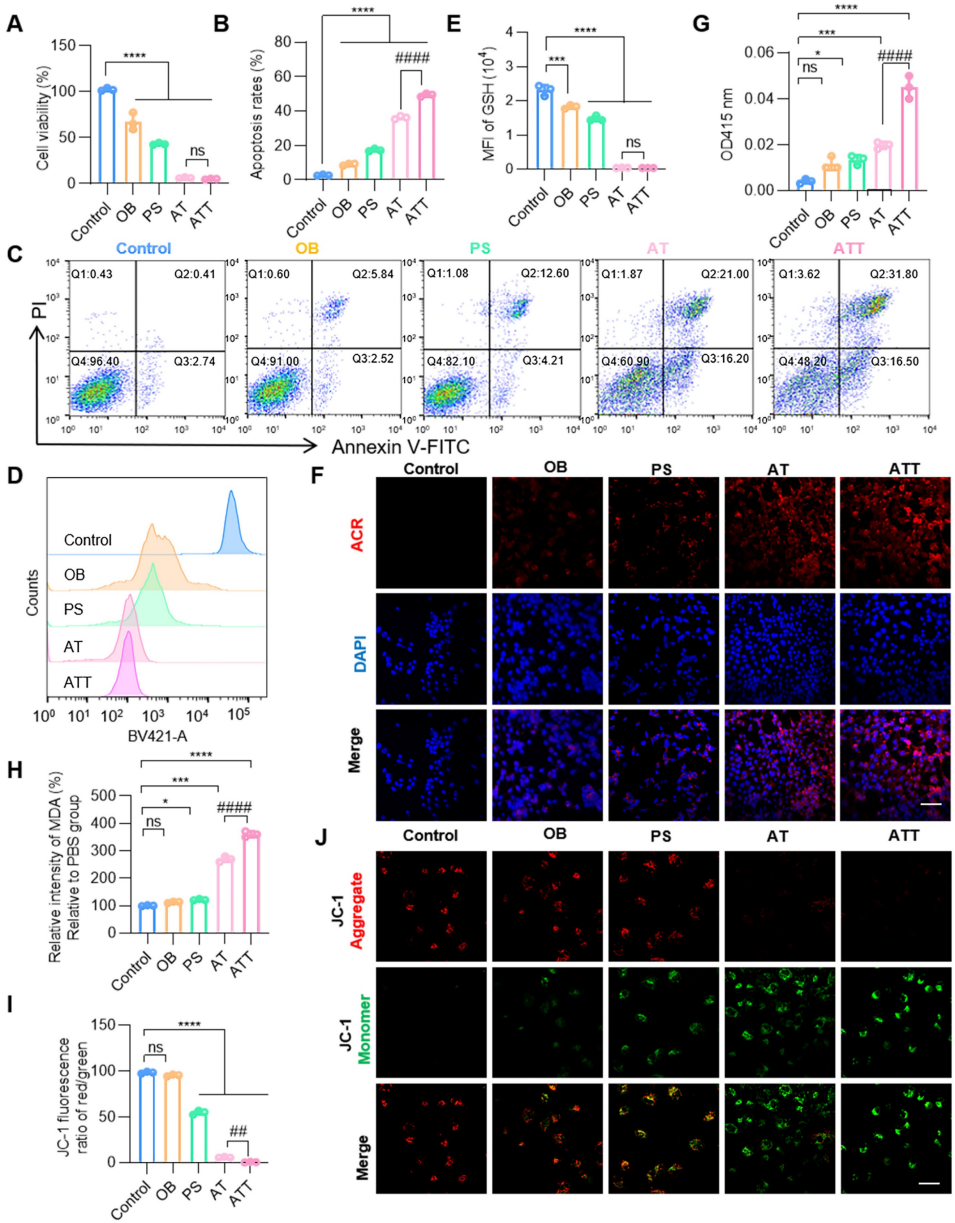
The influence of NPs on cell viability *in vitro*. A) Cell viability after different treatments for 24 h. B) Quantitative analysis of apoptosis in C. C) Apoptosis analysis of BC cells after different treatments for 24 h. D) Flow cytometry analysis of GSH levels in BC cells after different treatments for 24 h. E) Fluorescence intensity of GSH after treatment NPs in D. F) CLSM images of ACR staining after different treatments for 24 h (scale bar, 20 μm). G) Detection of H_2_O_2_ after different treatment for 24 h under a H_2_O_2_ assay Kit. H) Detection of MDA after different treatment for 24 h under an MDA assay Kit. I) Quantitative analysis of the JC-1 fluorescence ratio (red/green) in J. J) CLSM images of JC-1 staining in BC cells after different treatments for 24 h (scale bar, 20 μm). Data are presented as mean ± s.d. *p < 0.01; ***p < 0.001; ****p < 0.001; ##p < 0.001; ####p < 0.001 ns, not significant by one-way ANOVA test.

After successful construction of the NPs, the cytotoxicity was verified. Results showed PAOX-SPD-BSO system effectively inhibited BC cell proliferation, with ATT exhibiting a cell viability reduction of over 95%, and the concentration of SPD and BSO were 20 µM and 25 µM (Figure 3A), which were significantly lower than those used for non-delivery. Co-treatment with BSO, SPD, and PAOX induced markedly higher cell death rates in BC cells compared to individual treatments. PAs are naturally occurring cationic aliphatic alkylamines that have protonated amino groups at physiological pH levels. Therefore, PAs can interact with negatively charged cellular components, exacerbating cellular damage 33,34. The OB alone had negligible effects on cell morphology, while PS induced morphological alterations. The combination of BSO, SPD, and PAOX led to a substantial increase in cell mortality, confirmed by live/dead staining (Figure S6). As shown in Figure 3B-C, the OB treatment induced apoptosis in ~8.36%, whereas ATT resulted in ~16.81% apoptosis, and co-treatment with BSO elevated apoptosis to ~50.10%, suggesting that BSO enhances the accumulation and cytotoxicity of PA oxidation products for synergistic therapeutic effects.

ACR is the highest reactive endogenous aldehyde capable of inducing oxidative stress, DNA damage, and protein crosslinking to induce cell death 35. BSO treatment at increasing concentrations (0 ~ 1 mM) resulted in dose-dependent depletion of intracellular GSH, with 90% GSH remaining when BSO concentration is 25 µM (Figure S7). Cells treated with AT and ATT showed decreased GSH levels by flow cytometry (Figure 3D-E). CLSM imaging revealed a progressive increase in intracellular ACR fluorescence intensity in the NP-treated groups compared to PBS controls (Figure 3F). In addition to ACR, the toxic substances generated during the PA oxidation process also included H_2_O_2_, which lead to an increase in the lipid peroxidation product Malondialdehyde (MDA) 36. We detected an increase in H_2_O_2_ and MDA in cells treated with ATT (Figure 3G-H). Considering that both ACR and H₂O₂ can induce oxidative damage to mitochondria 10, we used JC-1 staining, a classical method for assessing mitochondrial membrane potential, to evaluate cellular response. A significant decrease in mitochondrial membrane potential was observed in cells treated with ATT (Figure 3I-J), indicating that the combination therapy induced mitochondrial dysfunction via accumulation of toxic metabolites. These findings suggest that ATT NPs impair cell viability through multiple pathways to eliminate cancer cells.

Cell stimulations also reshape the tumor microenvironment 11. ACR levels in the culture supernatant following various treatments were quantified via UV spectrophotometry and showed a positive correlation with concentration (Figure S8). Supernatants from PS, AT, and ATT treatments all displayed elevated ACR levels, consistent with full-spectrum UV absorption results. The effects of these conditioned media on cell viability were assessed, supernatants from ATT-treated cells significantly reduced BC cell proliferation and colony formation (Figure S8E-G). Transwell migration assays showed that supernatants from ATT also impaired BC cell migration, with minimal migration observed in cells treated with AT-conditioned media.

### Inducing apoptosis of the ATT NPs

**Figure 4:**
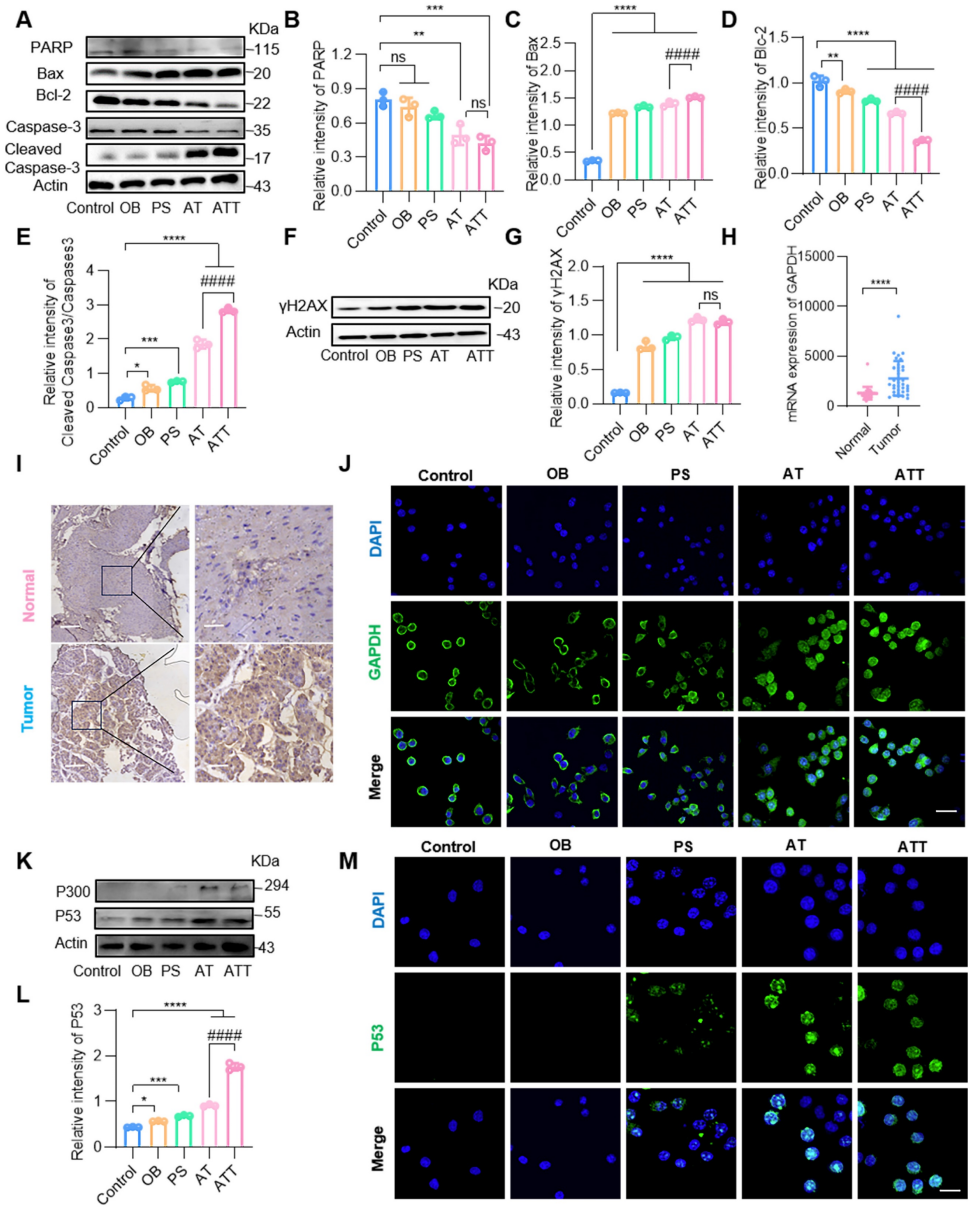
**NPs induced cell apoptosis through multiple mechanisms. (A)** Western blot analysis of PARP, Bax, Bcl-2, caspase-3 and cleaved caspase-3 expression in BC cells after different treatments for 24 h. Quantification of marker molecular PARP **(B)**, Bax **(C)**, Bcl-2 **(D)** and caspase3/cleaved caspase3 **(E)**. **(F)** Western blot analysis of γH2AX expression in BC cells after different treatments for 24 h. **(G)** Quantification of marker molecular γH2AX. **(H)** Quantification of GAPDH mRNA levels in human bladder cancer tissues and normal tissues in GEO database (GSE133624, 29 normal tissues and 36 tumor tissues). **(I)** GAPDH expression of bladder cancer tumor tissues and adjacent tissues. Scale bars in low magnification images represent 100 μm, and in high magnification images represent 20 μm. **(J)** CLSM images of GAPDH staining after different treatments for 24 h (scale bar, 20 μm). **(K)** Western blot analysis of P300 and P53 expression in BC after different treatments for 24 h. **(L)** Quantification of marker molecular P53. **(M)** CLSM images of staining for P53 after different treatments for 24 h (scale bar, 20 μm). Data are presented as mean ± s.d. *p < 0.01; **p < 0.01; ***p < 0.001; ****p < 0.0001; ####p < 0.0001 ns, not significant by one-way ANOVA test.

The combined drug delivery system killed cancer cells through multiple mechanisms. Therefore, a variety of apoptosis-related proteins were detected by Western blot. The results showed that Caspase-3, PARP and Bcl-2 levels decreased significantly, while Cleaved caspase-3 and Bax sharply increased in the AT and ATT groups (Figure 4A-E), indicating that the NPs were indeed involved in apoptotic activities. Both ACR and high concentrations of PAs bind to macromolecules such as nucleic acids and proteins, etc. 35,36, and cells treated with ATT showed decreased levels of multiple proteins, leading us to hypothesize that it affects protein expression. We extracted mRNA from cells treated with or without ATT and found that mRNA from cells treated with 10 μg ATT appeared more dispersed than that of 5 μg (Figure S9), suggesting that enhanced PA oxidation affects protein transcription, and consequently, protein translation. Additionally, we found that the DNA damage marker molecule γ-H2AX increased after treatment with NPs (Figure 4F-G).

Importantly, we found that GAPDH, an important enzyme in glycolysis, was decreased in cells treated with ATT, according to Western blot results (Figure S10A). As reported, GAPDH is not only an internal reference protein but also overexpressed in some cancer cells, through few studies focus on BC cells 37,38. Several databases showed that GAPDH expression levels in BC tissues were significantly higher than in normal tissues (Figure 4H, Figure S10B). Additionally, IHC results demonstrated that GAPDH was overexpressed in tumor cells (Figure 4I, Figure S10C), indicating that GAPDH might be a potential molecular target for the treatment and diagnosis of BC. ACR binds to various proteins including GAPDH, and the binding of ACR to GAPDH induces apoptosis, which is similar to the apoptosis caused by NO-mediated inactivation of GAPDH 39,40. Our results showed that ACR binding to GAPDH induced the translocation of the complex to the nucleus (Figure 4J), and the P300 level was significantly increased, suggesting that the P300/CBP pathway was activated. Next, we detected the downstream molecular target P53 of the P300/CBP pathway by Western blot and found that the expression of P53 was increased (Figure 4K-M). Thus, the P300/CBP/P53 pathway was activated, leading to apoptosis. These results indicate that ATT not only promotes the accumulation of ACR and binds to GAPDH to activate the P300/CBP/P53 pathway, thereby inducing apoptosis, but also reduces GAPDH content, blocking the carbohydrate metabolism pathway and inhibiting cell proliferation. Interestingly, MTT assay results showed that individual treatment with SPD, BSO and ACR were more toxic to epithelial cells than cancer cells, but had superior antitumor effects when combined (Figure S11). This might be because ACR, in addition to being primarily detoxified by GSH, also interacts with other protein molecules to weaken cytotoxicity 41,42. However, binding to overexpressed GAPDH in tumor cells further induces apoptosis, resulting in a better therapeutic effect on tumor cells. These findings suggest that ATT affects cell activity through multiple mechanisms, and its cytotoxicity depends on many synergistic effects.

Pifithirn-α hydrobromide (PH, a P53 expression inhibitor) 43 was used to investigate the effects of ATT@H on P53 expression. MTT assay demonstrated that the inhibitor reversed 80% of killing effect induced by ACR, indicating that P53 was important in inhibiting activation. Western blotting showed PH down-regulated P53 expression as well as the expression of downstream proteins of Blc-2 and Bax 44,45. After PH treatment, the expression of bcl-2 and Bax also reversed (Figure S12). This intriguing finding suggests that the activation state of P53 triggered by ATT@H exerts a significant influence on the regulation of apoptotic proteins.

### Inducing lipid peroxidation

**Figure 5:**
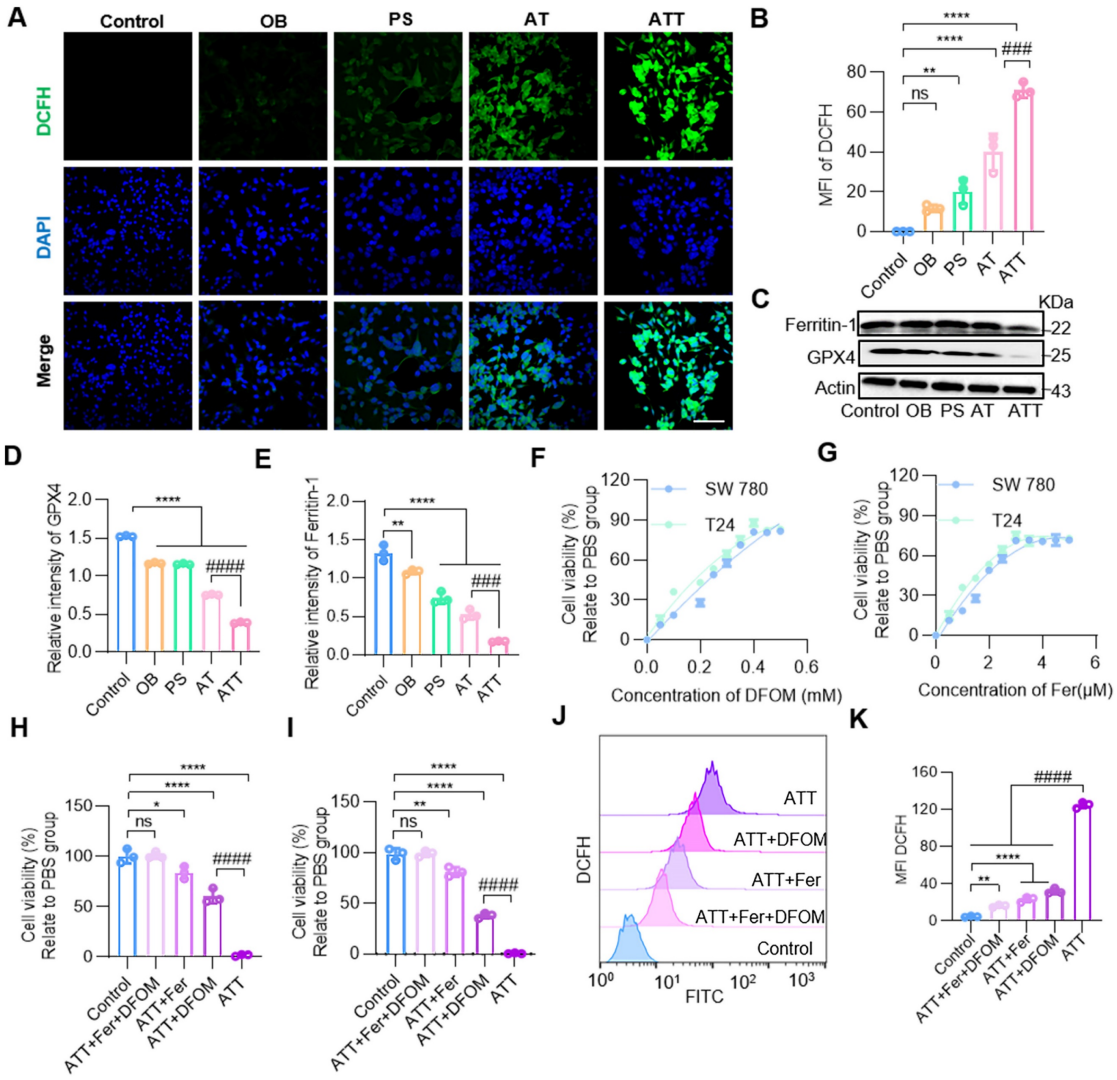
**NPs induced lipid peroxidation. (A)** Confocal images of staining for DCHF after different treatment for 24 h (scale bar, 20 μm). **(B)** Quantification of mean fluorescence intensity of DCFH in A. **(C)** Western blot analysis of ferritin-1 and GPX4 expression in BC cells after different treatments for 24 h. Quantification of marker molecular GPX4 **(D)** and ferritin-1 **(E)**. **(F)** Concentration of DFOM on cell viability. **(G)** Concentration of Fer on cell viability. **(H)** Cell viability effect of ferroptosis inhibitors on bladder cancer cells SW780. **(I)** Cell viability effect of ferroptosis inhibitors on bladder cancer cells T24. **(J)** Flow cytometry analysis of DCFH in ATT treated BC cells after incubation with ferroptosis inhibitors for 24 h. **(K)** Quantitative analysis of DCFH in J. Data are presented as mean ± s.d. *p < 0.01; **p < 0.01; ****p < 0.0001; ###p < 0.001; ####p < 0.0001; ns, not significant by one-way ANOVA test.

Metabolism requires mitochondria to supply energy; multiple metabolic pathways were inhibited after ATT treatment, and both ACR and H_2_O_2_ caused oxidative damage to mitochondria. JC-1 staining showed that the combination treatment damaged mitochondria through the accumulation of toxic substances. The H_2_O_2_ produced by PA oxidation further forms reactive oxygen species (ROS), which induce lipid peroxidation 46. Moreover, the contents of H_2_O_2_ and MDA increasing during the oxidation process of PA, suggesting cells were induced oxidative damage after treatment ATT NPs. And our results showed the fluorescence intensity of DCFH was significantly increased in the ATT group (Figure 5A-B).

Ferroptosis is a novel form of programmed cell death caused by the accumulation of iron-dependent lipid peroxides 47-49. Therefore, we further investigated whether the accumulation of PA metabolites could lead to ferroptosis. Western blot analysis showed that GPX4, a core regulator of ferroptosis, was decreased, and ferritin-1, a major iron storage protein complex in eukaryotic cells, was also decreased after treatment with NPs (Figure 5C-E). First, BC cell lines SW780 and T24 were treated with ATT, followed by the addition of different concentrations of the ferroptosis inhibitor ferrostatin-1 (Fer) and deferoxamine mesylate (DFOM), respectively. The results showed that cell viability gradually recovered as the concentration of ferroptosis inhibitor increased. The highest cell viability was reached approximately 60% with 5 μM Fer and approximately 80% with 0.4 mM DFOM (Figure 5F-G). Next, the highest concentrations of the two ferroptosis inhibitors were selected, and an MTT assay was performed. The results showed that cell viability increased after treatment, and the effect of the combination of Fer and DFOM was more significant than that of either used alone (Figure 5H-I). Then, cells treated with ATT and ferroptosis inhibitors were analyzed for DCFH production by flow cytometry. The results showed that DCFH levels decreased in the ferroptosis inhibitor-treated groups, and the DCFH level was lower than that with the ferroptosis inhibitor used alone (Figure 5J-K). These results indicate that the harmful metabolites of PAs promote ferroptosis through two mechanisms: lipid peroxidation and iron release.

To further investigate the underlying mechanism of ATT in BC, cells treated with or without ATT were collected and analyzed by metabolomics sequencing using LC-MS. A total of 28 upregulated and 272 down-regulated proteins were detected, involving more amino acids and nucleic acids. The 2-phosphoglyceric acid, a metabolite of glycolysis, was significantly increased in the ATT treatment group, indicating its downstream activity was inhibited. This suggests that the increase in ACR might affect the glycolysis process and interfere with cellular functions (Figure S13), although this needs to be further verified with experimental data. Both GSH and oxidized GSH levels were sharply reduced, suggesting that BSO and PAOX inhibited GSH synthesis, thereby disrupting the redox balance *in vivo*. After ATT treatment, various metabolic pathways were inhibited, including carbohydrate metabolism, amino acid metabolism, and lipid metabolism, with 50, 38 and 30 metabolites altered respectively (Figure S14). Together, these results show that the disruption of PAs metabolism affects other metabolic processes, particularly carbon metabolism and amino acid metabolism, indicating that it kills tumor cells through multiple mechanisms, including DNA damage, lipid oxidation, and cell apoptosis.

Moreover, the ATT@H group exhibited a statistically superior therapeutic efficacy compared to the ATT group at cellular levels, attributable to the hydrogel's dual role in sustained drug release and minimized systemic leakage (Figure S15). Besides GADPH, it has been reported that ACR activates and influences matrix metalloproteinase-9 (MMP-9), which plays important in regulating extracellular matrix remodeling including invasion, metastasis, angiogenesis and so on 50. We further investigated the MMP-9 expression after different treatment, and results showed ATT@H significantly increased the expression of MMP-9 (Figure S16), indicating that ATT@H had potential to modify the cellular microenvironment.

### Anti-tumor activity *in vivo*

**Figure 6:**
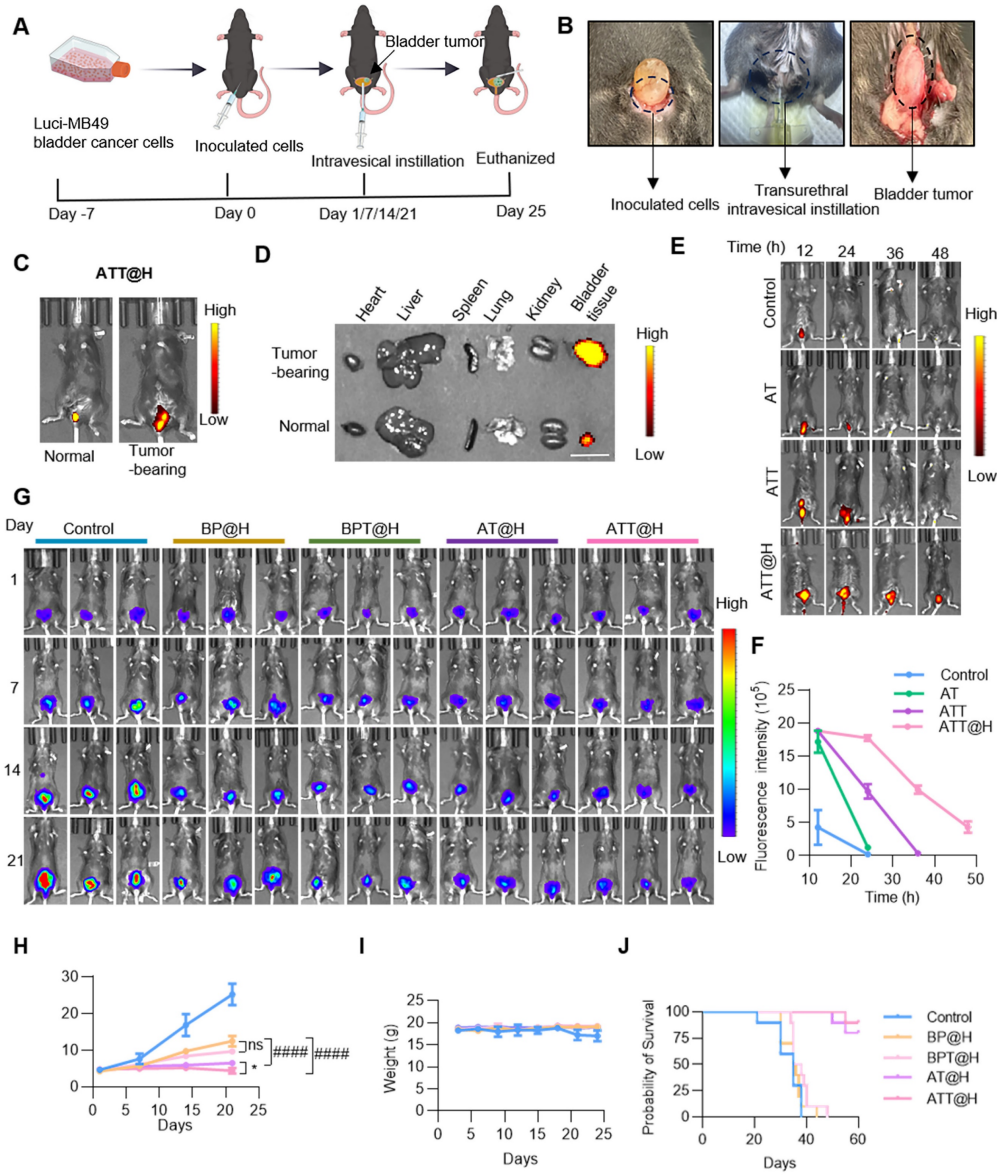
**Anti-tumor efficacy of NPs-hydrogel system. (A)** Diagram showing establishment orthotopic BC mouse model and the experimental procedure for evaluating the anti-tumor efficacy of the intravesical instillation NP-embedded hydrogel system. **(B)** Scheme of construction Orthotopic BC mouse model (left), transurethral intravesical instillation (medium) and taken out tumor (right). **(C)** Fluorescence intensity of ATT@H irrigated into normal mouse (left) and tumor bearing mouse (right). **(D)** Fluorescence intensity of different organs taken out from normal mouse and tumor bearing mouse. Scale bar indicates 1 cm. **(E, F)** Fluorescence images and quantitative analysis of fluorescence intensity of NPs with or without embedded hydrogel irrigated into BC tumor-bearing mice at different time points. **(G)** Representative bioluminescent images of BC tumor-bearing mice with different treatments (n = 5). **(H)** Quantitative analysis of bioluminescent intensity of different treatments in (G)**. (I)** Statistical analysis of body weight with different administrations **(J)** Kaplan-Meier survival analysis of BC mouse model treated with different administrations (n = 10). Data are presented as mean ± s.d. *p < 0.01; ####p < 0.0001; ns, not significant by one-way ANOVA test.

In previous studies, the effect on cell viability in the OB and PS groups was lower than that in the AT and AT groups, indicating the importance of targeting peptides. To investigate the effect of endogenous SPD on ACR production after different treatments, orthotopic BC mouse model was constructed and performed intravesical instillation therapy. The animals were divided into five groups, including Control group treated with PBS, BSO/PAOX-encapsulated MSNs (BP) in hydrogels (defines as BP@H), targeting peptide-modified BP in hydrogels (defines as BPT@H), AT in hydrogels (defines as AT@H) and ATT in hydrogels (defines as ATT@H) (Figure 6A-B). Firstly, to access the targeting performance *in vivo*, NPs were labeled with FITC and then perfused into the bladders of tumor-bearing and non-tumor-bearing mice. Results showed that the abdominal fluorescence intensity of tumor-bearing mice was significantly higher than that of non-tumor-bearing mice (Figure 6C), and the fluorescence intensity of the bladder tumor was significantly higher than that of the bladder and other organs (Figure 6D). These results indicated that Bld-1 promoted specific targeting of tumor tissues by the NPs with minimal effect on other organs. Next, Cy5-labeled hydrogel was used for bladder instillation, the fluorescence intensity of the hydrogels was quantified as a function of time. Figure S18 showed that the hydrogels remained *in vivo* over 72 h, indicating it had capacity to increase drug retention time in bladder. The hydrogel exhibited temperature-responsive behavior, being a gel state at 37 °C and undergoing a transition to a liquid state at room temperature. To investigate the effect of the hydrogel on drug release, the NPs were labeled with FITC and encapsulated with or without hydrogel, then perfused into BC mice for different time periods. As shown in Figure 6E-F, ATT demonstrated a 50% retention rate, whereas the non-targeted AT group exhibited near-complete clearance. The fluorescence intensity of tumors in ATT@H group was significantly higher than that in AT@H group. After tumors were prepared into paraffin sections, IHC staining showed PAOX content in ATT@H group was higher than that of in AT@H group, indicating the enhanced tumor cell penetration in the ATT@H group (Figure S17). The results suggested that the modification target peptide promoted rapid localization of NPs in tumors and significantly prolonged retention time *in vivo*. Moreover, ATT@H group retained ~23.03% in bladder over 48 h and ATT was undetectable at 36 h, indicating that the hydrogel enhanced NP adhesion to tumors and improved cellular uptake. These results suggest that the target peptide Bld-1 promotes targeting of tumor cells, and the hydrogel enhances the residence time *in vivo*, thereby achieving sustained drug release. All of the NPs were embedded in hydrogel and irrigated into the bladder of the MB49 BC mouse model, then visualized using the IVIS Lumina system. The results showed that ATT@H had the lowest fluorescence intensity among all groups (Figure 6G-H), which indicating the best inhibition of BC. Interestingly, the groups without exogenous SPD supplementation still exhibited an inhibitory effect on tumor growth, but it was lower than that of the supplementation group (Figure 6G), suggesting that endogenous PAs in BC also exhibited anti-tumor activity after PAs oxidation. This might be due to the disruption of amino acid synthesis and other metabolic pathways after drug administration, leading to the inability of PAs to be replenished through synthesis or transport, resulting in the depletion of the PAOX substrate. Therefore, the addition of exogenous SPD enhanced the anti-tumor activity. Moreover, except for the PBS group, the body weight of other groups showed no significant changes (Figure 6I). Benefiting from the remarkable anti-tumor effect, the mice with BC experienced prolonged survival time through the PAOX-SPD-BSO drug delivery system (Figure 6J).

### Safety evaluation of the drug delivery system

**Figure 7:**
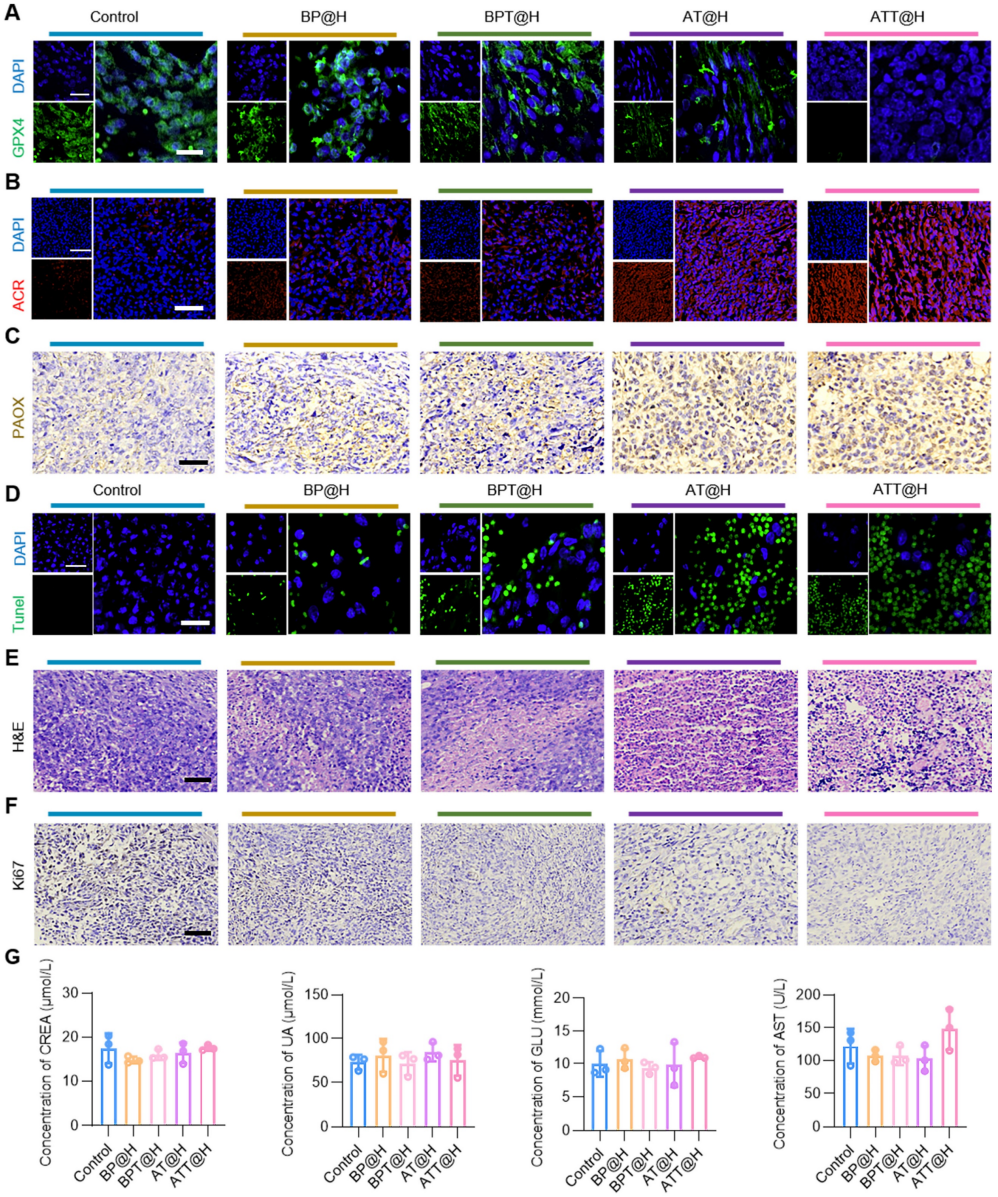
** Safety evaluation of the NPs irrigated into BC cell-bearing mouse. (A)** GPX4 staining in tumor tissues from different treatment groups after administration for 24 days (Scale bars: 20 μm for low magnification images and 50 μm for high magnification images). **(B)** ACR staining in tumor tissues from different treatments after administration for 24 days (Scale bars: 50 μm for low magnification images and 100 μm for high magnification images). **(C)** IHC analysis of PAOX staining in tumor tissues from different treatment groups after administration for 24 days (Scale bars: 50 µm). **(D)** TUNEL staining in tumor tissues from different treatments after administration for 24 days (Scale bars: 50 µm). **(E)** H&E staining in tumor tissues from different treatments after administration for 24 days (Scale bars: 50 µm). **(F)** IHC analysis of Ki67 staining in tumor tissues from different treatments after administration for 24 days (Scale bars: 50 µm). **(G)** Blood test results of different groups (n = 3). CREA, creatinine; GLU, glucose; UA, uric acid; AST, aspartate transaminase.

To further explore the *in vivo* mechanisms of the drug delivery system, tumor tissues were excised for IHC and immunofluorescence analyses. As shown in Figure 7A, GPX4 expression was the lowest in the ATT@H group, indicating extensive oxidative damage and confirming ferroptosis as a key mechanism of cell death *in vivo*. Post-treatment, ACR staining intensity increased significantly compared to the PBS group (Figure 7B), while PAOX expression was markedly elevated in PAOX-administered groups (Figure 7C), demonstrating that the drug system effectively facilitated ACR accumulation *in vivo*.

Encouraged by the notable *in vitro* and *in vivo* therapeutic effects, a comprehensive safety assessment was conducted. Extensive necrosis was observed in both ATT@H and BP@H groups, consistent with terminal deoxynucleotidyl transferase mediated dUTP nick-end labeling (TUNEL) results. Compared to NPs lacking Bld-1, peptide-functionalized formulations significantly enhanced anti-tumor activity (Figure 7D-E), confirming the role of Bld-1 in facilitating rapid cellular uptake and boosting therapeutic efficacy. Additionally, Ki67 staining showed a significant reduction in Ki67-positive cells in the ATT@H and BP@H groups relative to the PBS control (Figure 7F), indicating inhibited tumor cell proliferation. Importantly, no significant histopathological lesions were observed in major organs across all treatment groups (Figure S19), underscoring the low systemic toxicity and favorable biocompatibility of the NPs. As for the peripheral blood biochemical in indicators in different treatment groups, the results showed no significant changes in the levels of serum alanine transaminase (ALT), aspartate aminotransferase (AST), alkaline phosphatase (ALP), glucose (Glu), creatinine (CREA), uric acid (UA) and blood urea nitrogen (BUN) in mice (Figure 7G, Figure S20). Together, the above results demonstrate that SPD-mediated ACR not only exhibits superior anti-tumor activity, but also has excellent biocompatibility, making it a promising candidate for *in vivo* tumor therapy.

To investigate the safety of ATT@H delivery system and the potential application of combined traditional chemotherapy drugs, we conducted four groups, including Control, ATT, ATT@H and mitomycin C, for 6-week instillation therapy and 2-week drug withdrawal. The results showed therapeutic effect of ATT@H was significantly higher than ATT group (Figure S21), benefiting from the enhanced drug retention time in bladder by hydrogel. There were no damages or pathological abnormalities of the main organs including heart, liver lung, and kidney after different treatments according to the H&E staining on 6-week after treatments (Figure S22). Moreover, serum biochemical analysis of hepatic and renal function parameters suggested that ATT@H featured reliable biosafety. After 2-week drug withdrawal, bladder tumors in ATT@H groups exhibited a low proliferative activity, indicating a low recurrence rate. These results suggesting ATT@H had relatively fewer side effect.

Above all, ATT@H delivery system prolongs the retention time and facilitates its penetration into tumor cells by hydrogel, resulting in remarkable superior therapeutic effects compared to traditional chemotherapy drugs for long-term treatment. H&E staining and blood test showed low system toxicity high biocompatibility, making it a promising candidate for clinical application.

MSN is widely applied in the biomedical application such as oral drug delivery, bioimaging and photothermal therapy benefiting from its high biocompatibility. Various nano-drugs are entered the clinical trial stage and achieved astonishing results 51,52. Polyamine-targeting agents are currently undergoing clinical trials, especially the development of polyamine synthesis inhibitors 53, such as AMXT 1501 (a polyamine transport inhibitor) and Eflornithine (an ornithine decarboxylase 1enzyme inhibitor) 53,54. These ongoing clinical trials endorse the translational potential of the polyamine-based therapeutic strategy for BC treatment. ACR enhances oxidative stress, which might induce immunogenic cell death (ICD) 10,55. Various chemotherapy drugs function cytocidal effect through mechanisms of oxidative damage, exemplified by doxorubicin and cisplatin 56,57. ATT@H is predicted to synergize with chemotherapy, potentiating tumor cell killing by possibly triggering an immune response and boosting overall anti-tumor activity. Combination thermo-sensitive hydrogel remarkably prolongs drug retention time and enhances therapeutic effect. A comparative study was conducted on the therapeutic efficacy of ATT@H and traditional chemotherapy drug mitomycin C, result showed ATT@H exhibited superior anti-tumor activity than mitomycin C, especially in the long-term treatment. These results provide a broad prospect for clinical application. Certainly, to achieve clinical trials, toxicology, safety assessment, as well as pharmacokinetic exploration of ATT@H require further study.

## Conclusions

In summary, we developed a novel therapeutic approach for BC through constructing a NP-embedded reverse temperature hydrogel to product PAs-mediated the production of ACR and inhibit its detoxification via GSH, effectively enhancing ACR accumulation both *in vitro* and *in vivo*. This strategy led to a loss of mitochondrial membrane potential and altered the expression of key apoptosis-related proteins such as Caspase-3, PARP, Bax and Bcl-2. Additionally, the treatment induced lipid peroxidation and down-regulated GPX4 and Fer. ACR also elevated γH2AX expression, indicating DNA damage, and promoted GAPDH binding and promoting its nuclear translocation. ACR induced lethal carbonyl stress, disrupted redox balance *in vivo*, and killed tumor cells in multiple mechanisms. Besides, the reverse temperature hydrogel was initially in a liquid state and the gelated *in situ* to promote uniform NPs dispersion throughout the bladder and sustain drug release. Altogether, our findings demonstrate that enhancing intracellular ACR accumulation represents a promising strategy for cancer therapy.

## Materials and Methods

### Materials

8-PEG-CHO was purchased from ShangHai ToYongBio Tech. Inc. SPD, BSO and RPMI 1640 medium were purchased from Sparkjade Scientific Instruments Co, Ltd. Annexin V/PI Apoptosis Detection Kit was purchased from Vazyme Biotech, Co Ltd. PAOX polyclonal antibody was purchased from Proteintech Group, Inc. JC-1 was purchased from Yeasen Biotechnology (Shanghai) Co., Ltd. MDA detection Kit and H_2_O_2_ detection kit were purchased from Solarbio Life Sciences. The other chemicals and regents were highest quality available.

### Cell lines, animals and clinical samples

BC cell lines MB49-luci and SW780 were incubated in RPMI 1640 supplemented with 10% fetal bovine serum (FBS). Bladder epithelial cells SV-HUC-1 was incubated in Ham's F-12K supplemented with 10% FBS. The 4~6 weeks C57 mice weighting 18.0 to 20.0 g were raised in comfortable environment, fed standard food, and allowed to drink freely. All animal experiments were approved by the Animal Care and Experiment Committee of Qilu Hospital affiliated to Shandong University (approval No. DWLL-2024-150). The clinical samples about BC tumor and adjacent tissue were provided by Qilu Hospital of Shandong University. All human samples experiments were implemented according to the Medical Ethics Committee of Shandong University Qilu Hospital (Document numbers KYLL-202506-011-1).

### Prepare of PPP polymers

Poly(ethylene glycol) (Mn = 6000 Da, 4.01 g) was dehydrated at 130 °C under vacuum, followed by argon purging. At 100 °C, lactide (7.06 g), glycolide (2.27 g), and Sn(Oct)₂ (12 mg in toluene) were added. After solvent removal, polymerization proceeded at 150°C for 12 h under argon. Unreacted monomers were eliminated by vacuum distillation, and the product was purified via triple water rinsing and lyophilization, with final storage at -20 °C. ^1^H NMR measurements were performed in D_2_O on a 400 MHz Avance III HD spectrometer (Bruker, Germany).

### Preparation of PPP-based hydrogels

For the preparation of PPP hydrogels, the synthesized PPP polymer (15%, w/v) was dissolved in PBS solution under vortexing at 25 ℃. The mixture was homogenized by stirring, and then self-assembled into gel state at 37 °C. For the preparation of the NP-embedded hydrogels, the PPP solution (15%, w/v) was mixed with the suspension of MSN-based nanoparticles (40 μg/μL) and then allowed to stand statically at 37°C.

### Preparation of NPs

2 mg/mL MSN incubated with 0.10 µg/mL SPD and 0.15 µg/mL BSO by electrostatic adsorption in PBS solution at room temperature for 4 h, and then coated 0.35 µg/mL PAOX by electrostatic adsorption in PBS solution at room temperature for 2 h followed by centrifugation to remove the unbound component. 0.1 µg/mL Bld-1 was grafted with AT via 0.1 µg/mL 8-PEG-CHO. Every step followed by centrifugation to remove the unbound component. Finally, the different NPs were distributed in PBS for further use respectively. For detection of flow cytometry and CLSM, the NPs were incubated with FITC in PBS for 2 h at 4 °C, and then followed by centrifugation to remove FITC.

### Antitumor activity of NPs *in vivo*

Orthotopic BC mouse model was constructed as previous reported. Briefly, the mice were anesthetized and then inoculated with luciferin-expressing MB49 cells into the bladder wall using insulin syringes. The treatment commenced on day 10 post-inoculation, when mice exhibiting a mean fluorescence intensity of 45,000 ± 3,000 a.u. were selected to minimize inter-animal variability. After model establishment, the mice were divided into five groups randomly (n = 5) and treated regularly. The mice were treated with different formulations of NPs hydrogel hybrid system (200 mg/kg NPs in 50 μL of 15% hydrogel, 150 mg/mL) for bladder irrigation. The tumor dimension and body weight were recorded before injecting the drugs within 21 days of first administration. After intraperitoneally injected D-luciferin, the mice were anesthetized, and then the tumor burdens and fluorescence intensity were measured by IVIS spectral imaging system. The images were analyzed by the Living Image software. After the administration, the mice were euthanized and the tumors or organs were isolated for following experiments.

A detailed description of all materials and methods used is provided in *Supporting information*.

### Statistical analysis

Statistical analyses were performed using Origin 8.0 and SPSS version 23. Each experiment was done at least three times by triplicates. For comparison the statistical differences between tween groups, unless otherwise stated, Student's t-text (two-text) was carried out. Asterisks indicate significant difference (*p < 0.05, **p < 0.01, ***p < 0.001, ****p < 0.0001); Pound signs indicate significant difference between AT and ATT groups (#p < 0.05, ##p < 0.01, ###p < 0.001, ####p < 0.0001), p < 0.05 was considered statistically significant.

## Supplementary Material

Supplementary materials and methods, figures.

## Abbreviations

PAs: polyamines
SPD: spermidine
ACR: acrolein
IHC: immunohistochemistry
BSO: DL-Buthionine-(S,R)-sulfoximine
NPs: nanoparticles
BC: bladder cancer
MIBC: muscle-invasive bladder cancer
GSH: glutathione
ROS: reactive oxygen species
PAOX: polyamine oxidase
MSN: mesoporous silica nanoparticles
FBS: fetal bovine serum
MTT: methyl thiazolyl tetrazolium
PVDF: polyvinylidene difluoride
DFOM: malondialdehyde
Fer: ferrostatin-1
8-PEG-CHO: 8-arm-poly(ethylene glycol)-benzaldehyde
