# Supplementary material for: Targeted Accumulation of Reactive Acrolein via Nanoparticle-embedded Reverse Temperature Hydrogel for Bladder Cancer Treatment
Source: Theranostics. 2026 Jan 1;16(3):1528–44. doi: 10.7150/thno.119307 (PMC12679701; doi:10.7150/thno.119307)
Supplement: Supplementary file 1 — Supplementary materials and methods, figures. [file thnov16p1528s1.pdf]

Targeted accumulation of reactive acrolein via nanoparticle-embedded reverse temperature hydrogel for bladder cancer treatment

Yingying Xu[a,1], Qinglong Du[a,b,1], Zhongwei Zhao[a,1], Shuai Fu[c,1], Aijing Zhang[a,b], Jianguo Zheng[a,b], Chengyang Zhao[a,b], Yuxiang Meng[a,b], Hanru Liu[d], Zongyao Lv[d], Xin Qin[a,*], Huimin Geng[d,*], Nengwang Yu[a,*]

[a] Department of Urology, Qilu Hospital of Shandong University, Jinan 250012, China

[b] Cheeloo College of Medicine, Shandong University, Jinan 250012, China

[c] Department of Medical Oncology, Shandong Cancer Hospital and Institute, Shandong First Medical University and Shandong Academy of Medical Sciences, Jinan 250012, China

[d] Department of Neurosurgery, Qilu Hospital of Shandong University, Institute of Brain and Brain-Inspired Science, Shandong University, Jinan 250012, China

***Corresponding Author**

Nengwang Yu, qiluyunengwang@hotmail.com; Huimin Geng, hmgeng@sdu.edu.cn; Xin Qin, qinxinsdu2018@163.com.

[1]These authors contributed equally to this work.

Contents

SECTION1: Supplementary methods 3

1. Prepare of MSN. 3

2. Characterizations of NPs 3

3. Rheological measurements. 3

4. Release of encapsulation component *in vitro*. 3

5. Drug release behaviors of NP-hydrogel systems. 4

6. The targeting of Bld-1 to bladder were carried out by flow cytometry. 4

7. Cell apoptosis. 4

8 MTT assay. 4

9. Western Blot assay. 4

10. Flow cytometry assay. 5

11. Confocal laser scanning microscopy analysis 5

12. Mitochondrial membrane potential. 5

13. Live & Dead cells staining. 5

14. Cell morphology. 5

15. Cell migration. 5

16. Colony formation assay. 5

17. H_2O_2 assay. 6

18. MDA assay. 6

19. Acrolein assay. 6

20. Glutathione assay. 7

21. Immunohistochemistry. 7

22. Degradation of the hydrogel in bladder. 7

SECTION 2: Figure S1-S22. 9

SECTION1：Supplementary methods.

1. Prepare of MSN. The synthesis of MSN was initiated by dissolving cetyltrimethylammonium toluene sulfonate (CTAT, 0.96 g) in 50 mL of deionized water under continuous stirring. Triethanolamine (174 mg) was subsequently introduced into the mixture, resulting in the formation of a milky colloidal dispersion. Following a 60-minute stabilization period to ensure homogeneity, tetraethyl orthosilicate (TEOS, 7.81 mL) was slowly introduced into the system to commence the silica polymerization reaction. The homogeneous solution was then transferred to a PTFE-coated reaction vessel and subjected to hydrothermal treatment at 80°C for 2 h to facilitate controlled crystallization. The obtained MSNs were thoroughly rinsed with alternating water and ethanol cycles to remove residual surfactants, dehydrated under reduced pressure at 80 °C, and finally thermally treated in a muffle furnace at 550°C for six hours to eliminate organic templates.

2. Characterizations of NPs. The nanoparticle size and zeta potential were analyzed by a DLS (Zetasizer ZS90, Malvern Instruments), the experiment was replicated three times and averaged.

3. Rheological measurements. As for the method of rheological test, a rheometer (HAAKE RS6000, Germany) with a cone–plate (C35/1° Ti L07116, diameter 35 mm, and core angle 1°) was used to detect the rheological characterize of the hydrogel. The sample at 4 °C was placed on a parallel plate with an initial temperature of 25 °C. The changes in storage modulus (G′) and loss modulus (G") of the hydrogel were measured at a heating rate of 0.5 °C/min within the temperature range of 25 °C to 50 °C and the frequency at 1 Hz.

4. Release of encapsulation component *in vitro*. To investigate the release rate of encapsulation, we constructed OB, OS and OP as above mentioned in 2.3. BSO and SPD have the highest absorption peaks at 202 nm and 200 nm respectively, and is positively correlated with concentration within a certain range, which meant that SPD and BSO could be quantitatively test. The 25 μg/mL OB and 25 μg/mL OS were incubated at 37 °C for different interval (30 min, 1 h, 2 h, 4 h, 6 h, 8 h), respectively. At each point-in-time, the supernatant was centrifuged for full-wavelength scanning detection, and the released content was calculated

according to the drawn standard curve. As for PAOX, 50 μg/mL OP was incubated at 37 °C for different interval (30 min, 1 h, 2 h, 4 h, 6 h, 8 h), and the supernatant was centrifuged for protein content detection by BCA Protein AssayKit (Solarbio).

5. Drug release behaviors of NP-hydrogel systems. We initially synthesized drug-loaded NPs and, in parallel, fabricated drug-loaded NPs encapsulated within PPP hydrogels, respectively. All samples were then placed in PBS, and supernatant samples were collected at predetermined time points. BSO and SPD were detected by full-wavelength scanning, and PAOX was detected by BCA Protein AssayKit (Solarbio).

6. The targeting of Bld-1 to bladder were carried out by flow cytometry. Cells treated with or without ATT at 37 °C for different interval (30 min, 1 h, 2 h, 4 h, 6 h, 8 h), and the cells were collected and disperse in PBS for fluorescence intensity detection by flow cytometry analysis.

7. Cell apoptosis. Cells were seeded in 6-well plates and treated differently. After washing, the single-cell were obtained by digestion with EDAT-free tyrisin and incubated with Annexin V and PI successively according to instruction. Finally, cell apoptosis was detected by flow cytometry.

8 MTT assay. The cell proliferation of different treatment was detected by MTT assay. Cells were seeded in 96-well plate at a density of 3,000 cells/well and grown in appropriate cell culture medium. After treatment appropriate time, the cells were replaced by 100 μL fresh appropriate culture medium with final concentration of 0.5 mg/mL methyl thiazolyl tetrazolium (MTT), and were incubated at 37 ℃ for another 4 h. After removed the medium, formazan crystals were dissolved in 100 μL/well DMSO. The absorbance was measured at 570 nm by a microplate reader (BioTEK, USA).

9. Western Blot assay. Cells were treated differently, and then lysed in lysates containing cocktail on ice. The supernatant was obtained by centrifuged at 12,000×g, and mixed with loading buffer. After boiled for 10 min, the samples were separated by 10% SDS-PAGE and transferred to a polyvinylidene difluoride (PVDF) membrane (Millipore, USA). The samples

were incubated block buffer, primary and secondary antibody successively, and then visualized by enhanced chemiluminescence reagents.

10. Flow cytometry assay. Cells were treated differently, and then digested according to different conditions, cells were collected, and after washing by PBS, cells were performed flow cytometry.

11. Confocal laser scanning microscopy analysis. To visualize the fluorescence image, cells were seeded on seeded on poly-L-lysine–treated coverslips, and the treated different. After the treatment, cells were immobilized with 4% paraformaldehyde, washed by PBS, and treated different antibody or fluorescence dyes as needed. The nucleus was stained with DAPI for 15 min at room temperature. The slips were visualized by a scanning laser microscope.

12. Mitochondrial membrane potential. The mitochondrial membrane potential was analyzed by JC-1(5, 5′, 6, 6′-tetrachloro-1, 1′, 3, 3′-tetraethylbenzimi- dazolylcarbocyanine iodide) staining. Cells were seeded on Nucn EasYDish, and then treated differently. After incubating for 24 h, cells were washed by PBS, and stained with JC-1 staining at 37℃ for 15 min. Cells were observed by a confocal microscope.

13. Live & Dead cells staining. Cells were seeded in 24-well plates, and then treated differently. After incubating for 24 h, cells were incubated with Calcein-AM and PI for 30 min. After washing three times by PBS, cells were visualized by a fluorescence microscope.

14. Cell morphology. Cells were seeded in 24-well plates, and then treated differently. After incubating for 24 h, the cell morphologies were visualized by a microscope.

15. Cell migration. The cell migration experiment was carried out by transwell assay with 8-μm pores (Corning Inc., Corning, NY, USA). Cells were collected by trypsin digestion and seeded into the upper chambers of 10,000 cells/well and treated differently, and then placed in 24-well plates with 200 μL fresh medium every well. After incubating overnight, the upper chambers were washed, stained by 0.1% crystal violet staining solution, and analyzed by a microscope.

16. Colony formation assay. Cells were seeded in 6-well plates with 1,000 cells/well, and then treated differently. The wells were replaced with fresh medium with different treatment

respectively. After incubating for two weeks, the wells were fixed with 4% paraformaldehyde for 10 min, and the stained by 0.1 crystal violet staining solution after washed three times by PBS. The clones were counted.

17. H_2O_2 assay. The content of H_2O_2 in cells was detected by a H_2O2 Content assay kit (Solarbio, Beijing, China). Cells were seeded on 6-well plates, and then collected by centrifugation at 1,500 g for 5 min. Cells were resuspended in 1 mL regent I and disrupted by sonication with 30 repetitions per 3s in ice-water bath environment. After centrifugation at 8,000g for 10 min, at 4 °C, the supernatants were added reagents II, III, and IV successively according to the instruction. The productions were transferred to 96-well plates and analyzed at 415 nm using a microplate reader.

18. MDA assay. The content of malondialdehyde (MDA) in cells reflected the level of lipid oxidation in cell membranes. To investigate the effect of ATT on lipid oxidation, a micro-malondialdehyde (MDA) assay kit (Solarbio, Beijing, China) was used. Cells were seeded on 6-well plates, and then collected by centrifugation at 1,500 g for 5 min. Cells were resuspended in 1 mL extracting solution and disrupted by sonication with 30 repetitions per 3s in ice-water bath environment. After centrifugation at 8,000g for 10 min, at 4 °C, the supernatants were added reagents I, II, III, and IV successively and then kept warm at 100 °C for 60 min according to the instruction. The productions were transferred to 96-well plates and analyzed at 532 nm and 600 nm using a microplate reader. The content of MDA was calculated according to the instruction.

19. Acrolein assay. The ACR in cells were detected by full-wavelength scanning and ultraviolet spectrophotometry. ACR had the maximum absorption speak at 220 nm and is positively correlated with concentration within a certain concentration range. Cells were seeded on 96-well plates, and treated differently. After incubating for 24 h, the supernatant was collected and carried full-wavelength scanning. The difference in absorption peaks at 220 nm showed the produce of ACR.

To further analysis the production of ACR, an ultraviolet spectrophotometry was used. Firstly, ACR absorption solution was prepared by H_2O, ethanol-free aldehyde and HCl 1:2.5:0.5. Cells

were seeded on 6-well plates, and then treated differently. After incubating for 24 h, cells were resuspended in 1 mL PBS and disrupted by sonication with 30 repetitions per 3s in ice-water bath environment. After centrifugation at 8,000g for 10 min at 4 ℃, taken 100 μL of supernatants were added 1.9 mL of ACR absorption solution, 200 μL of tryptophan absorption solution (0.2% tryptophan in 0.1 M HCl) successively. After keeping warm at 50 ℃ for 30 min, the productions were analyzed at 570 nm by an ultraviolet spectrophotometer. ACR was prepared in ACR absorption solution at 0~1 μg/mL to draw standard curve.

20. Glutathione assay. The content of GSH in cells was detected by a reduced GSH content assay kit (Solarbio, Beijing, China). Cells were seeded on 6-well plates, and then collected by centrifugation at 1,500 g for 5 min. Cells were resuspended in 1 mL regent I and disrupted by sonication with 30 repetitions per 3s in ice-water bath environment. After centrifugation at 8,000g for 10 min, at 4 ℃, taken 20 μL of supernatants were added reagents II, and III successively according to the instruction. The productions were transferred to 96-well plates and analyzed at 412 nm using a microplate reader.

21. Immunohistochemistry. For H&E staining, the organs were fixed with 4% paraformaldehyde, and embedded in paraffin, sliced into 5 μm sections, and histologically examined for hematoxylin and eosin staining. For immunohistochemical staining, the sections were deparaffinated, rehydrated and boiled in 0.01 M Tris-EDTA pH 9.0 buffer. The sections were dyed with PAOX rabbit polyclonal antibody and incubated at 4 ℃ overnight. After washed by PBS, sections were added HRP-anti Rabbit antibody for 30 min. After thoroughly washed by PBS, sections were colored by DAB color solution for 5 min, and then washed with distilled water to terminate color development. Finally, hematoxylin redyed staining was performed. The colored slices were dehydrated, sealed, and then imaged by an orthotopic microscope.

22. Degradation of the hydrogel in bladder. To visually monitor the degradation process of hydrogels in vivo, Cy5-labeled PPP polymer was used to prepare PPP hydrogels. The hydrogels (50 μL) were injected subcutaneously into bladder of 6-week-old C57/BL6J mice (Charles river, China). At predetermined time points, all animals were imaged with an in vivo

imaging system (IVIS) Spectrum Instrument (PerkinElmer, USA). The fluorescence semi-quantitative analysis was carried out with the Living Imaging software.

SECTION 2: Figure S1-S22.

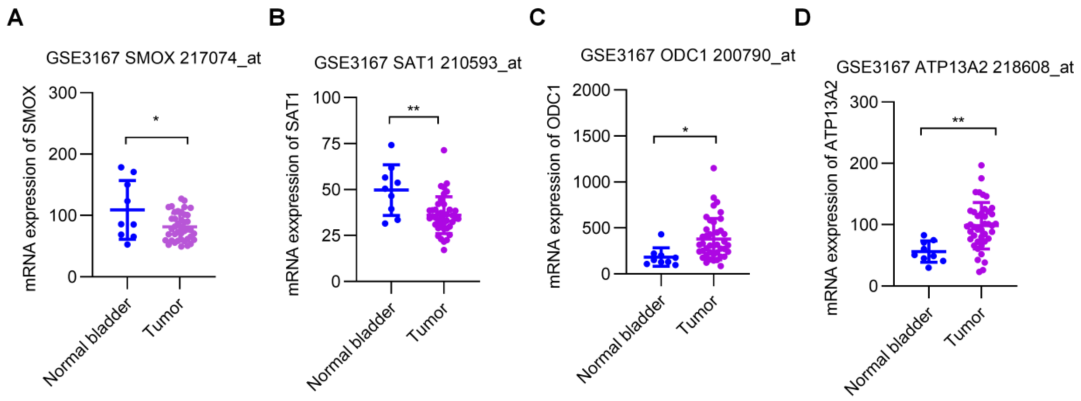

Figure S1. mRNA expression level of polyamine-relative metabolic enzymes and proteins in human bladder cancer tissues (n = 41) and normal tissues (n = 9) in GEO database (GSE3167). *p < 0.01; **p < 0.01 by one-way ANOVA test.

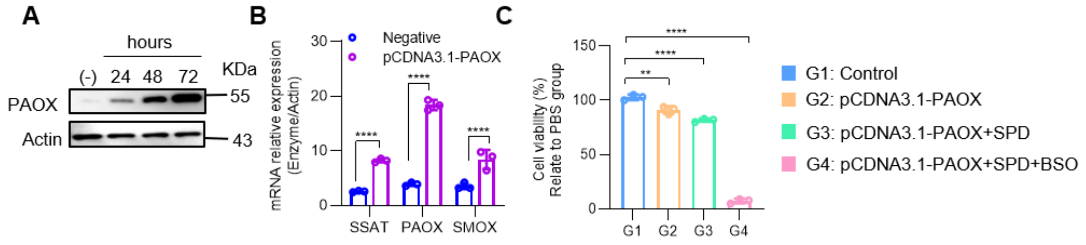

Figure S2. Effect of PAOX, BSO and SPD on cell viability. A) Western blot analysis of PAOX expression level after pCDNA3.1-PAOX plasmid transfected into bladder cancer cells for different times. B) RT-qPCR analysis of SSAT, PAOX and SMOX expression after transfected pCDNA3.1-PAOX into bladder cancer cells. C) Cell viability of PAOX, BSO and SPD in bladder cancer cell. G1, Control; G2, pCDNA3.1-PAOX; G3, pCDNA3.1-PAOX + SPD; G4, pCDNA3.1 + SPD+BSO. Data are presented as mean ± s.d. **$p < 0.01$; ****$p < 0.0001$ by one-way ANOVA test.

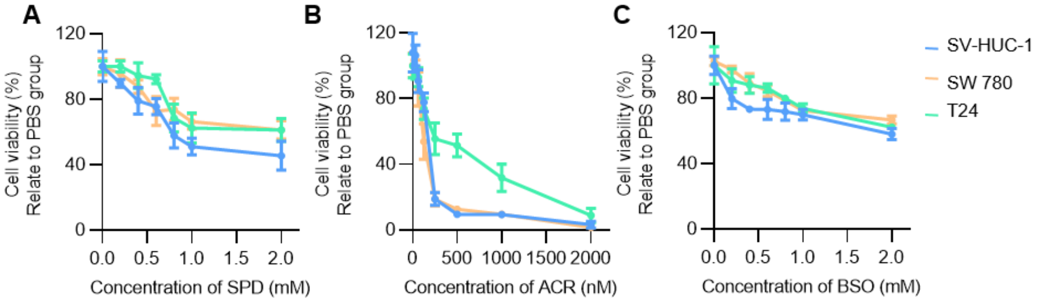

Figure S3. Cell viability of SPD (A), ACR (B) and BSO (C) on sw780, T24 and SV-HUC-1 cells.

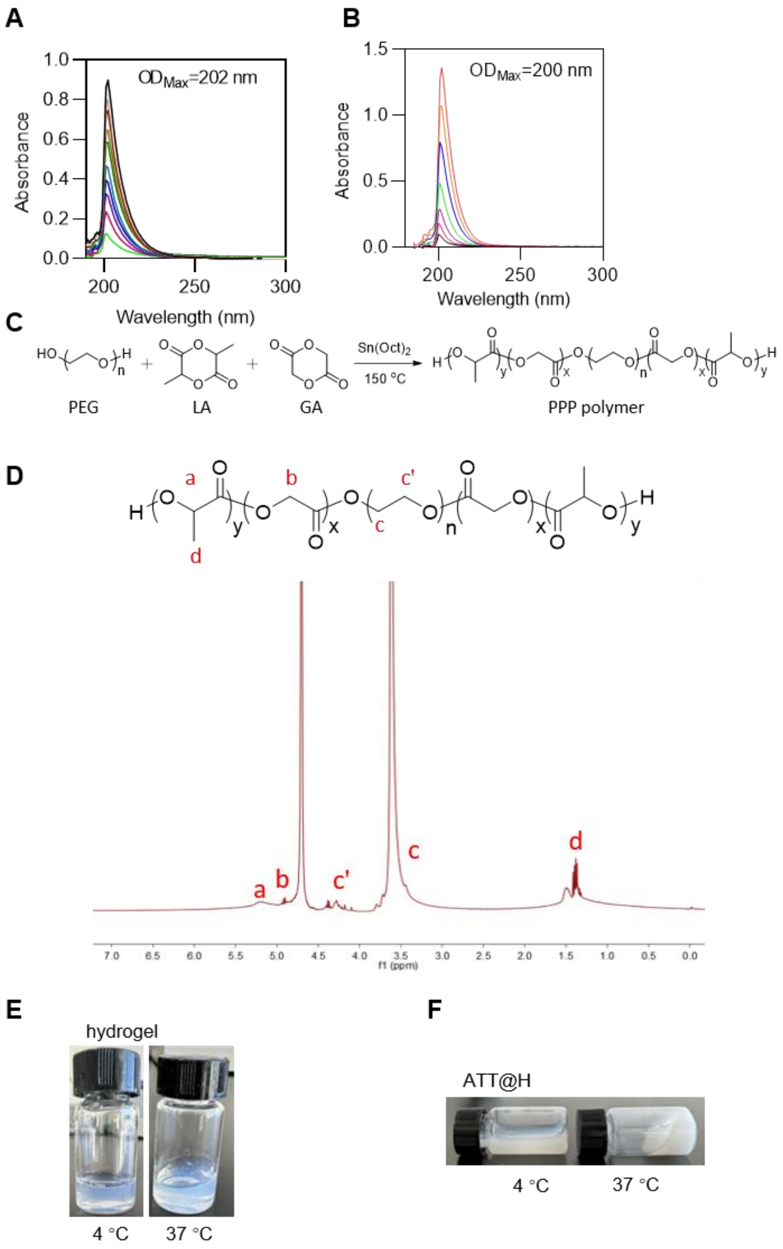

Figure S4. Characterized of nanoparticles. A) Full wavelength scanning of different concentration of BSO, from bottom to top: 0,0.01, 0.1, 0.15, 0.2, 0.25, 0.3, 0.35, 0.4, 0.45 and 0.5 μg/mL. B) Full wavelength scanning of different concentration of SPD, from bottom to top: 0, 0.05, 0.1, 0.15, 0.2, 0.25, 0.3 and 0.35 μg/mL. C) Diagram showing the PPP polymer composed of PEG, LA and GA to prepare temperature-responsive hydrogels. D) ^{1}HNMR spectra of PPP. E) Images showing the hydrogel at 4℃ (left) and 37 ℃ (right). F) Images showing the ATT@H at 4 ℃ (left) and 37 ℃ (right).

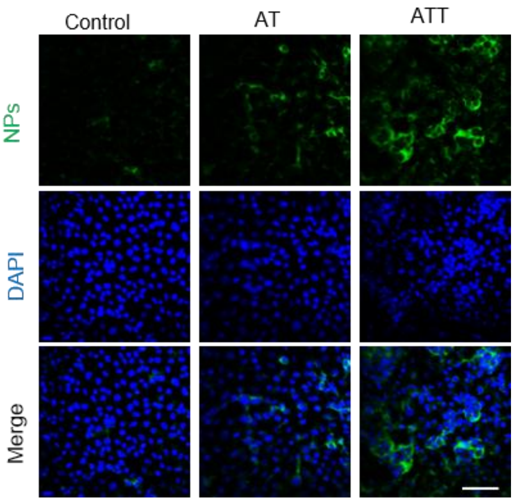

Figure S5. Cellular uptake of nanoparticles with or without Bld-1 by bladder cancer cells MB49 after incubated for 2h. Nanoparticles-FITC, green; Nucleus, blue. Scale bar indicates 20 μm.

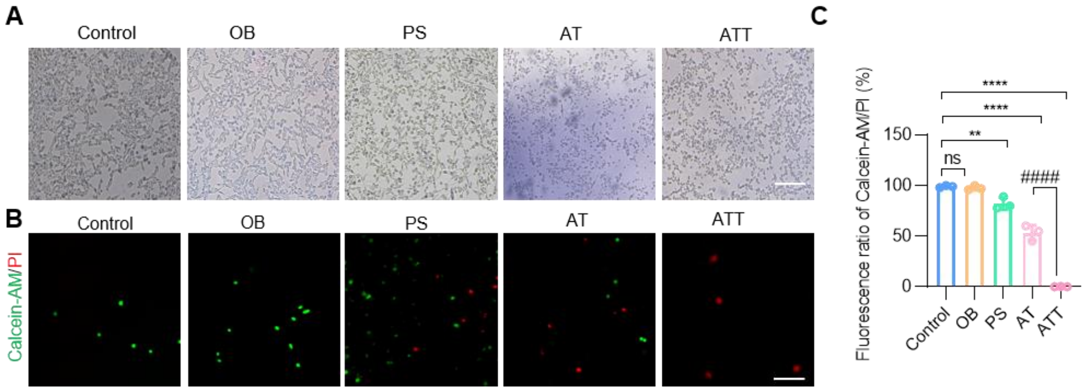

Figure S6. Effect of nanoparticles on cell viability. A) Cell morphology images of bladder cancer cells with different treatment for 24 h. Scale bar indicates 100 μm. B) Fluorescence images of bladder cancer cells in calcein-AM/PI staining with different treatment for 24 h. Scale bar indicates 100 μm. C) Quantification of fluorescence ratio of Calcein-AM/PI in B. Data are presented as mean ± s.d. **p < 0.01; ****p < 0.0001; ####p < 0.0001; ns, not significant by one-way ANOVA test.

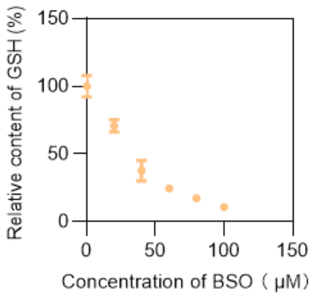

Figure S7. Effect of BSO concentration on GSH expression level.

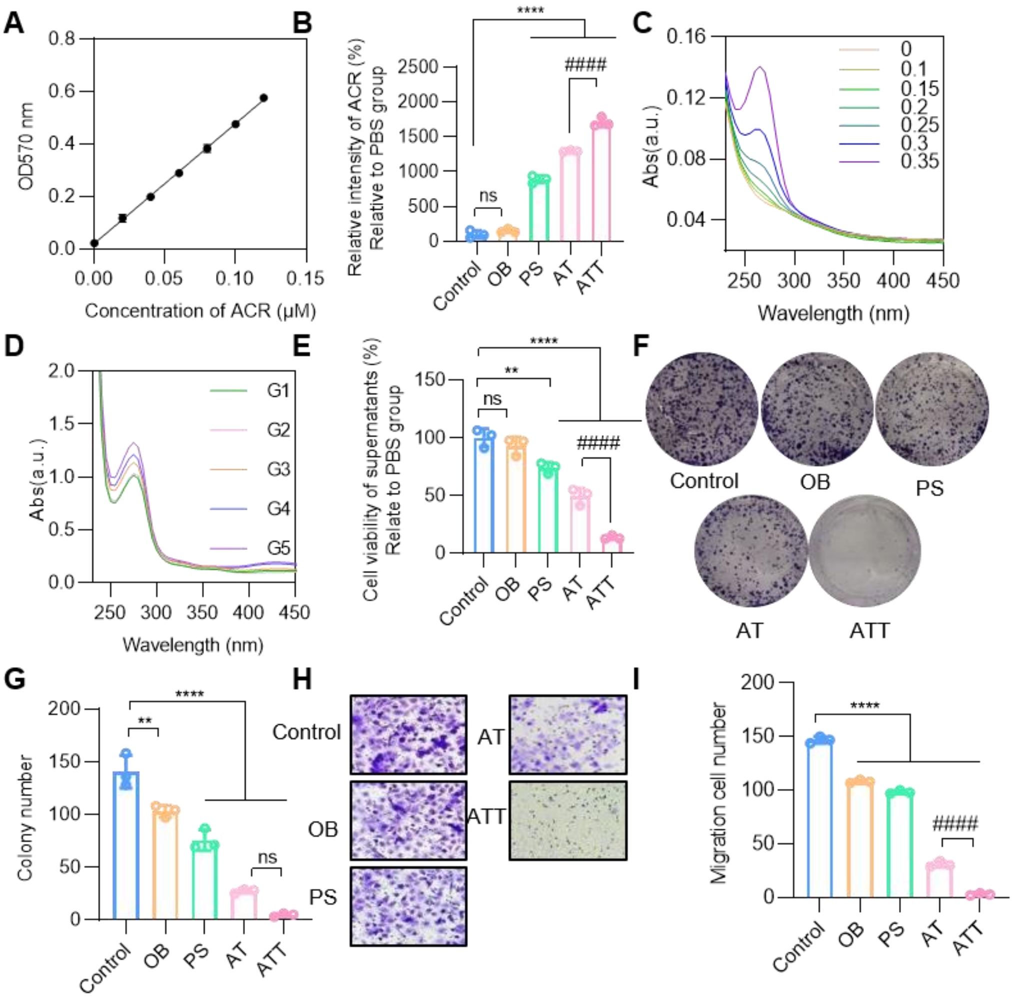

Figure S8. Effect of supernatants of different treatment on bladder cancer cell SW 780 viability. A) Difference concentration of ACR in the medium was detected by an ultraviolet spectrophotometry method. B) ACR content in supernatants of different treatment for 24 h. C) Full wavelength scanning of different concentration ACR, from bottom to top: 0,0.1, 0.15, 0.2, 0.25, 0.3 and 0.35 μg/mL. D) Full wavelength scanning of bladder cancer cells supernatants with different treatment for 24 h. E) Cell viability of supernatants of different treatment for 24 h. F) Colony number of bladder cancer cells with different treatment for 24 h. G) Quantification of colony number in F. H) Migration cell number of bladder cancer cells with different treatment for 24 h. I) Quantification of migration cell number in H. Data are presented as mean ± s.d. **$p < 0.01$; ***$p < 0.001$; ****$p < 0.0001$; ####$p < 0.0001$; ns, not significant by one-way ANOVA test.

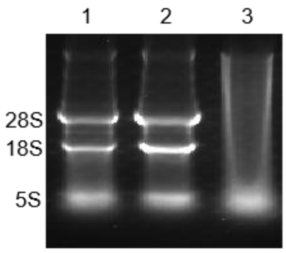

Figure S9. Effect of ATT on the amounts of 18S RNA and 28S RNA in bladder cancer cells. Lane 1, PBS; Lane 2, cells treated with 5 μg ATT; Lane 2, cells treated with 10 μg ATT.

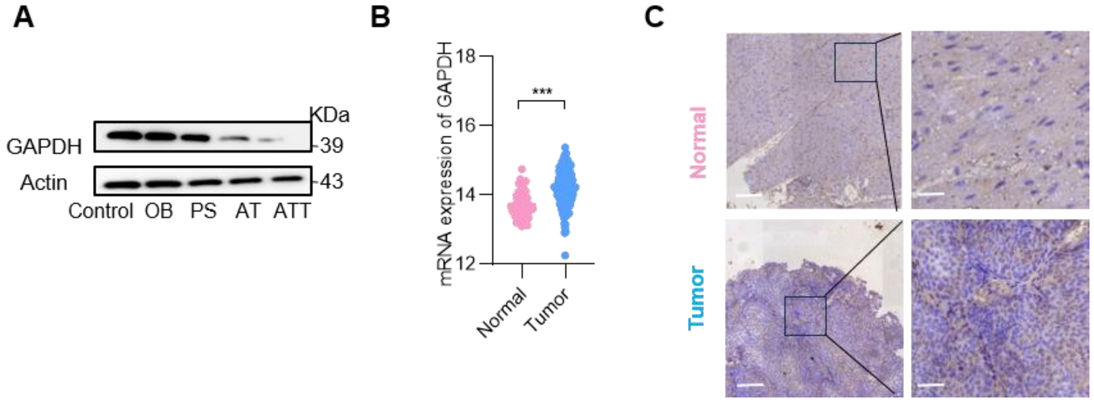

Figure S10. Analysis of GAPDH expression in bladder cancer. A) Western blot analysis of GAPDH expression in bladder cancer after different treatment for 24 h. B) Quantification of GAPDH mRNA levels in human bladder cancer tissues and normal tissues in GEO database (GSE13507, right, 68 normal tissues and 188 tumor tissues). C) GAPDH expression of bladder cancer tumor tissues and adjacent tissues. Scale bars in low magnification images represent 100 µm, and in high magnification images represent 20 µm. ***$p < 0.001$ by one-way ANOVA test.

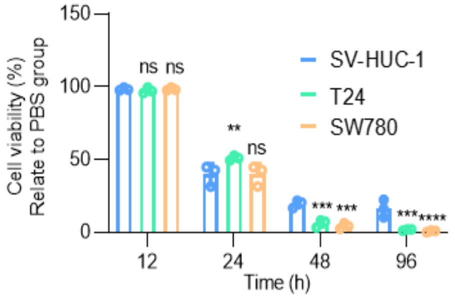

Figure S11. Cell viability of ATT treated for 96 h on SV-HUC-1, T24 and SW780 cells. Data are presented as mean ± s.d. $**p < 0.01$; $***p < 0.001$; ns, not significant by one-way ANOVA test.

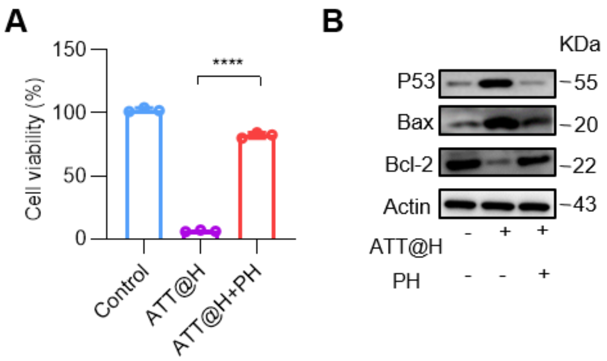

Figure S12. Effect of P53 on the therapeutic effect of ATT@H delivery systems. A) Cell viability after different treatments for 24 h. B) Western blot analysis of P53, Bax and Bcl-2 expression in BC cells after different treatments for 24 h. ****p < 0.0001 by one-way ANOVA test.

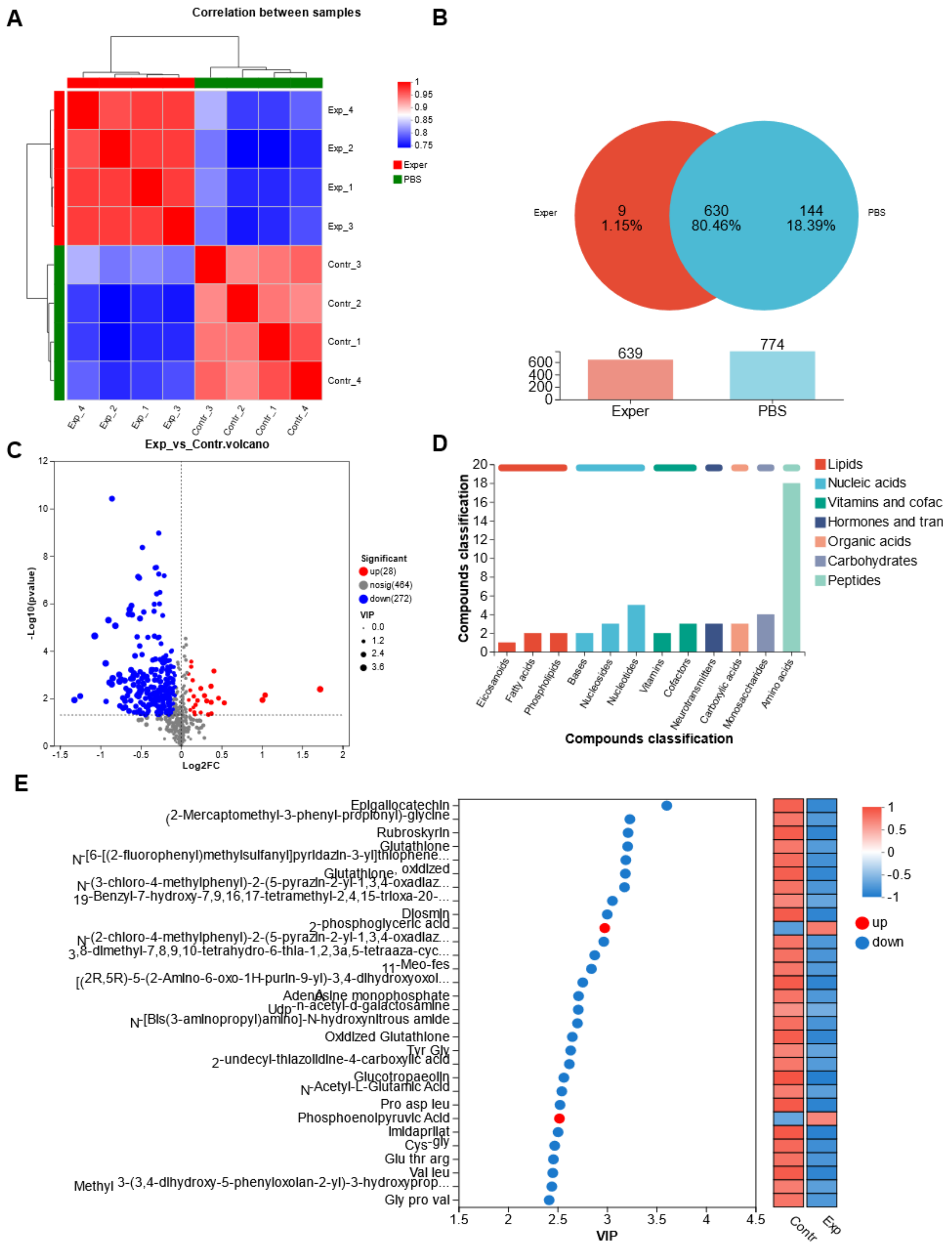

Figure S13. Metabolomics analysis of bladder cancer cells after treated ATT. A) Heatmap of correlation between ATT treated and PBS group. B) Venn diagram of sample size between ATT treated and PBS group. C) The amounts of molecular changed between ATT treated and PBS group. D) The amounts of metabolites changed

between ATT treated and PBS group. E) KEGG pathways changed between ATT treated and PBS group. ATT and PBS group were in quadruplicate.

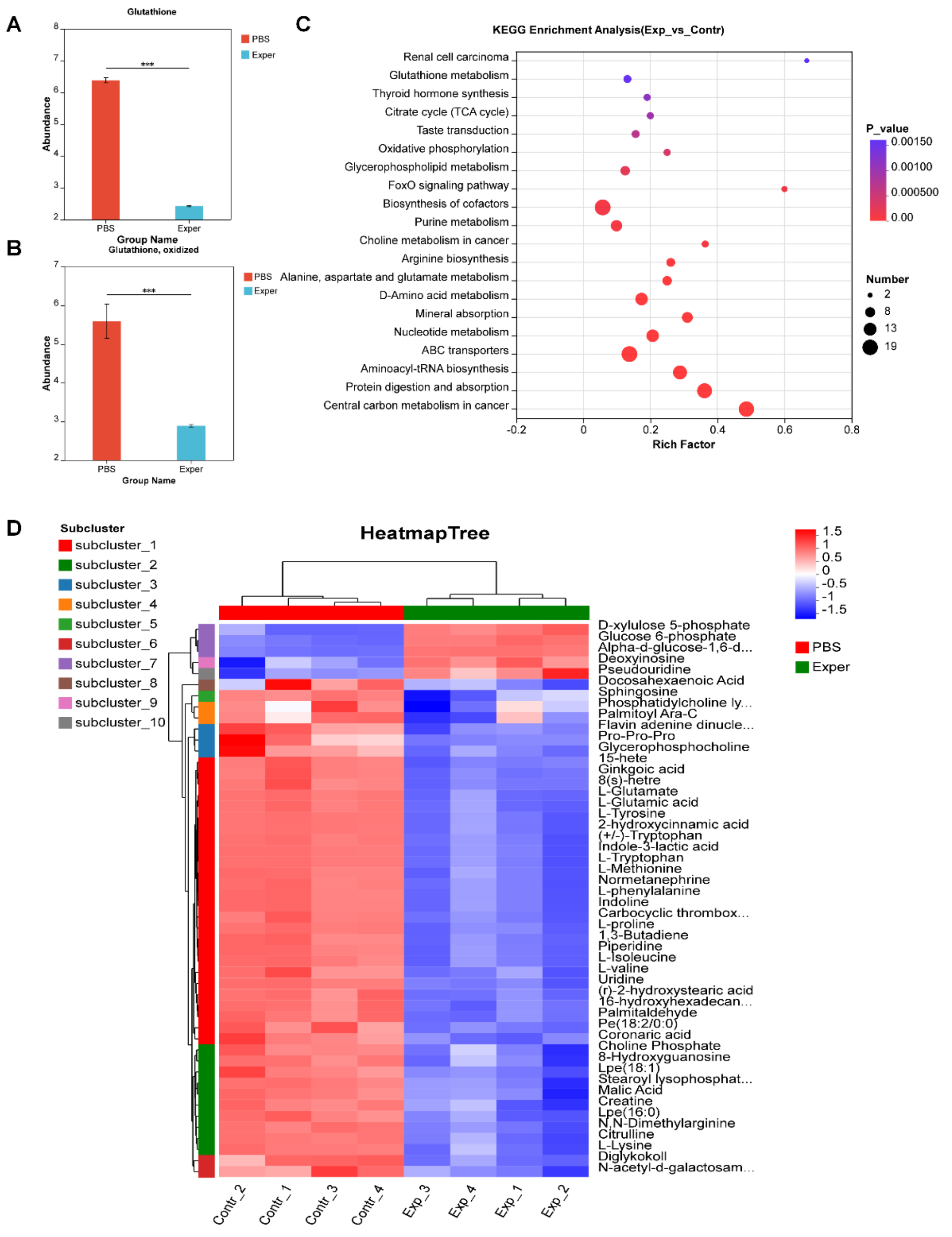

Figure S14. A) Expression of GSH. B) Expression of oxidized GSH. C) KEGG enrichment analysis. D)Heatmap of cluster analysis of metabolites

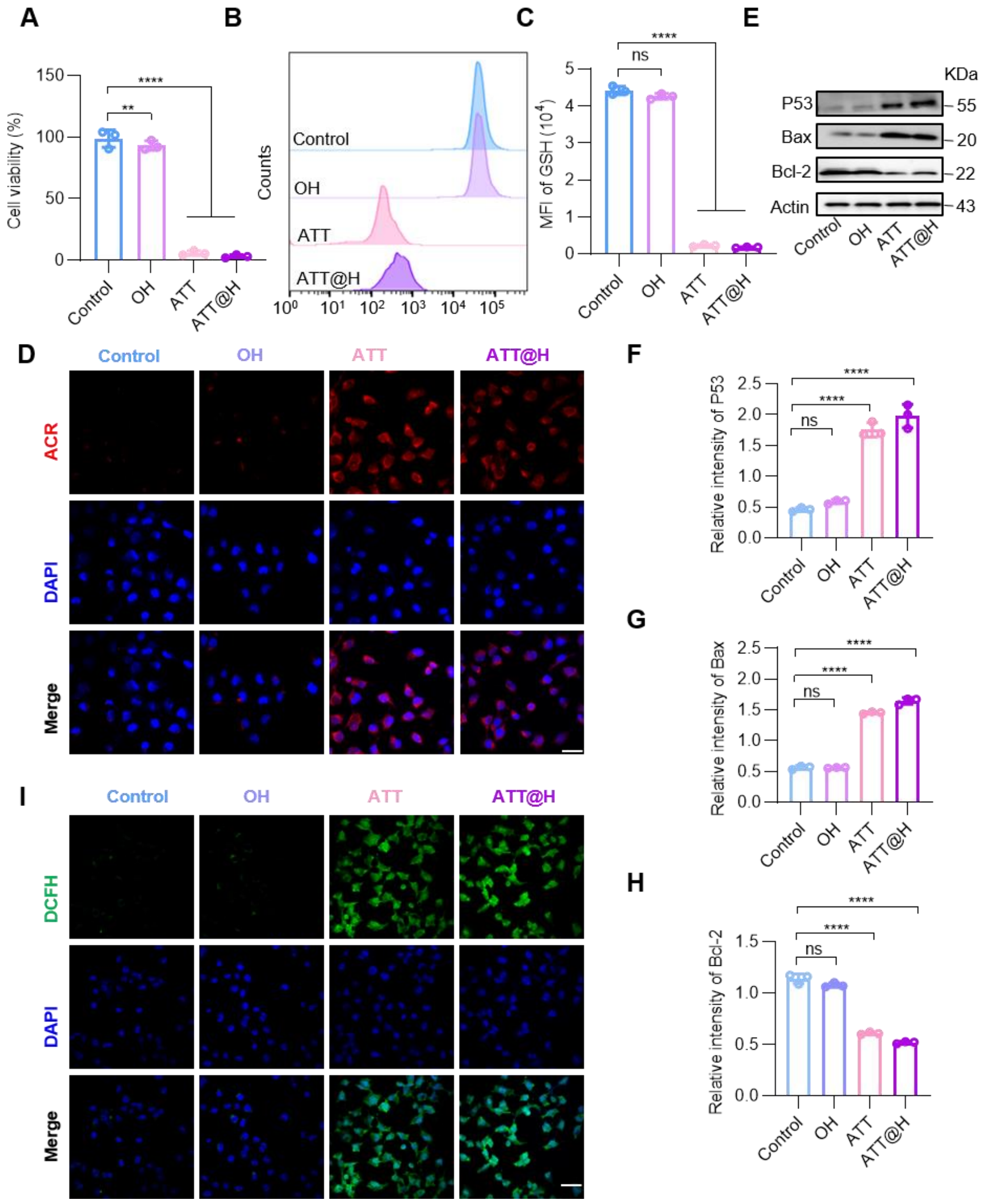

Figure S15. (A) Cell viability of SW780 cells with different treatments after 24 h. B) Flow cytometry analysis of GSH levels in BC cells after different treatments for 24 h. C) Fluorescence intensity of GSH after treatment NPs in B. D) CLSM images of ACR staining after different treatments for 24 h (scale bar, 50 μm). E) Western blot analysis of P53, Bax and Bcl-2 expression in BC cells after different treatments for 24 h. Quantification of marker molecular P53 (F), Bax (G) and Bcl-2 (H). I) Confocal images of staining for DCHF after different treatment for 24 h (scale bar, 50 μm). ****p < 0.0001; ns, not significant by one-way ANOVA test.

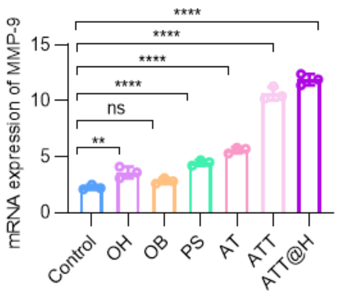

Figure S16. Quantification of MMP-9 mRNA levels in bladder cancer cells with different treatment. Data are presented as mean ± s.d. **p < 0.01; ****p < 0.0001; ns, not significant by one-way ANOVA test.

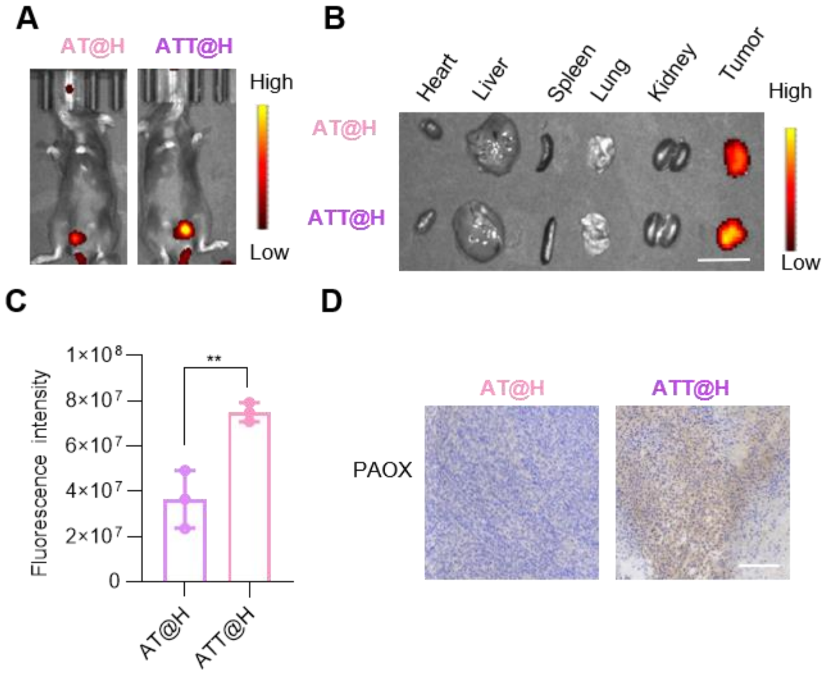

Figure S17. Targeting effect of peptide *in vivo*. A) Fluorescence image of AT@H (left) and ATT@H (right) irrigated into tumor bearing mouse. B) Fluorescence intensity of different organs taken out from tumor bearing mouse. Scale bar indicates 1 cm. C) Fluorescence intensity of tumors in B. D) IHC of PAOX staining for tumors in B (Scale bars: 50 μm).

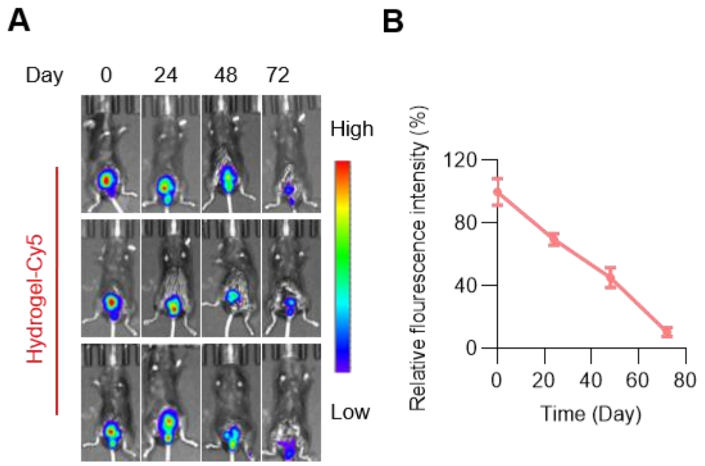

Figure S18. Real-time in vivo images (A) and fluorescence analysis (B) of the residual Cy5-labeled hydrogel in mice after bladder instillation.

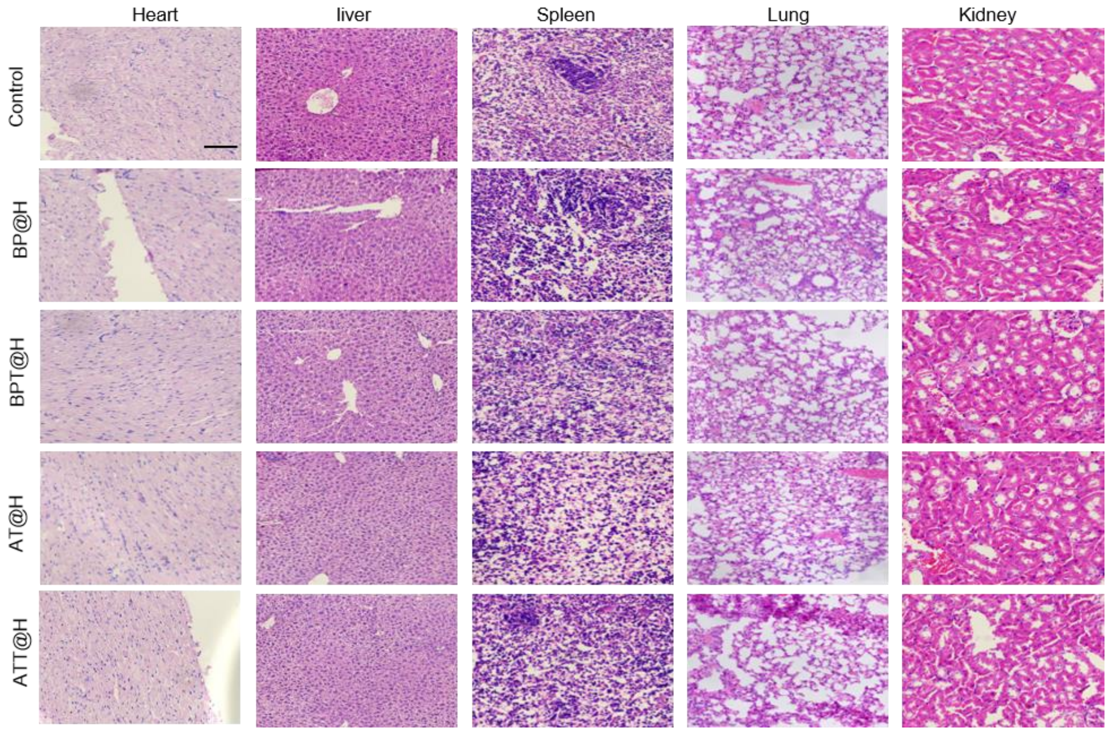

Figure S19. H&E staining of major organs in different treatments at 24 days (Scale bars are 50 μm).

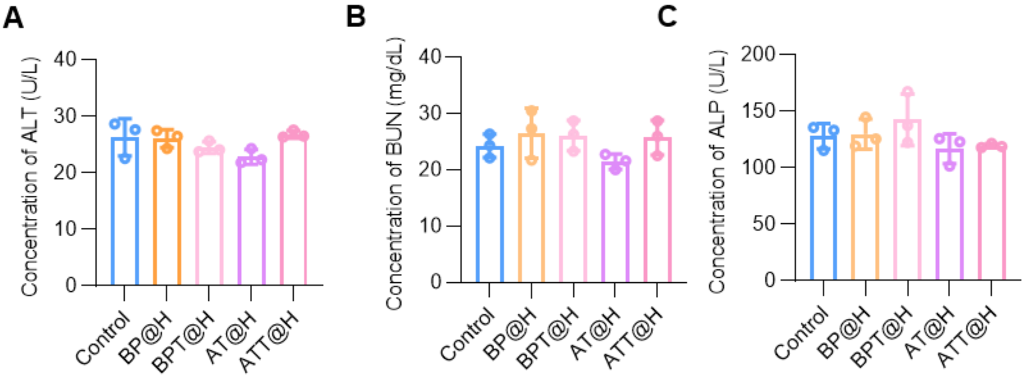

Figure S20. Blood test results of ALT (A), BUN (B) and ALP (C) in different groups (n = 3). ALT, alanine aminotransferase; ALP, alkaline phosphatase; BUN, blood urea nitrogen.

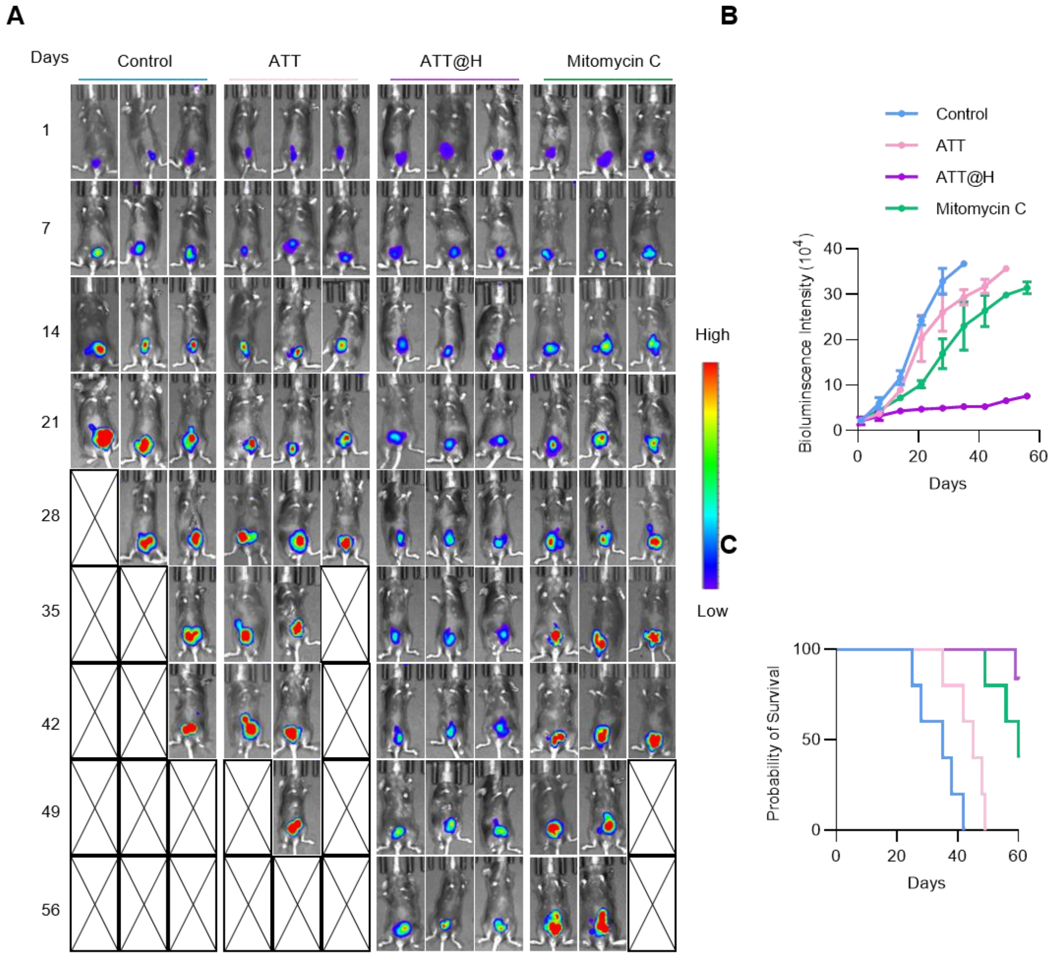

Figure S21. A, B) Fluorescence images and quantitative analysis of fluorescence intensity of NPs with or without embedded hydrogel irrigated into BC tumor-bearing mice at different time points. C) Kaplan-Meier survival analysis of BC mouse model treated with different administrations (n = 5).

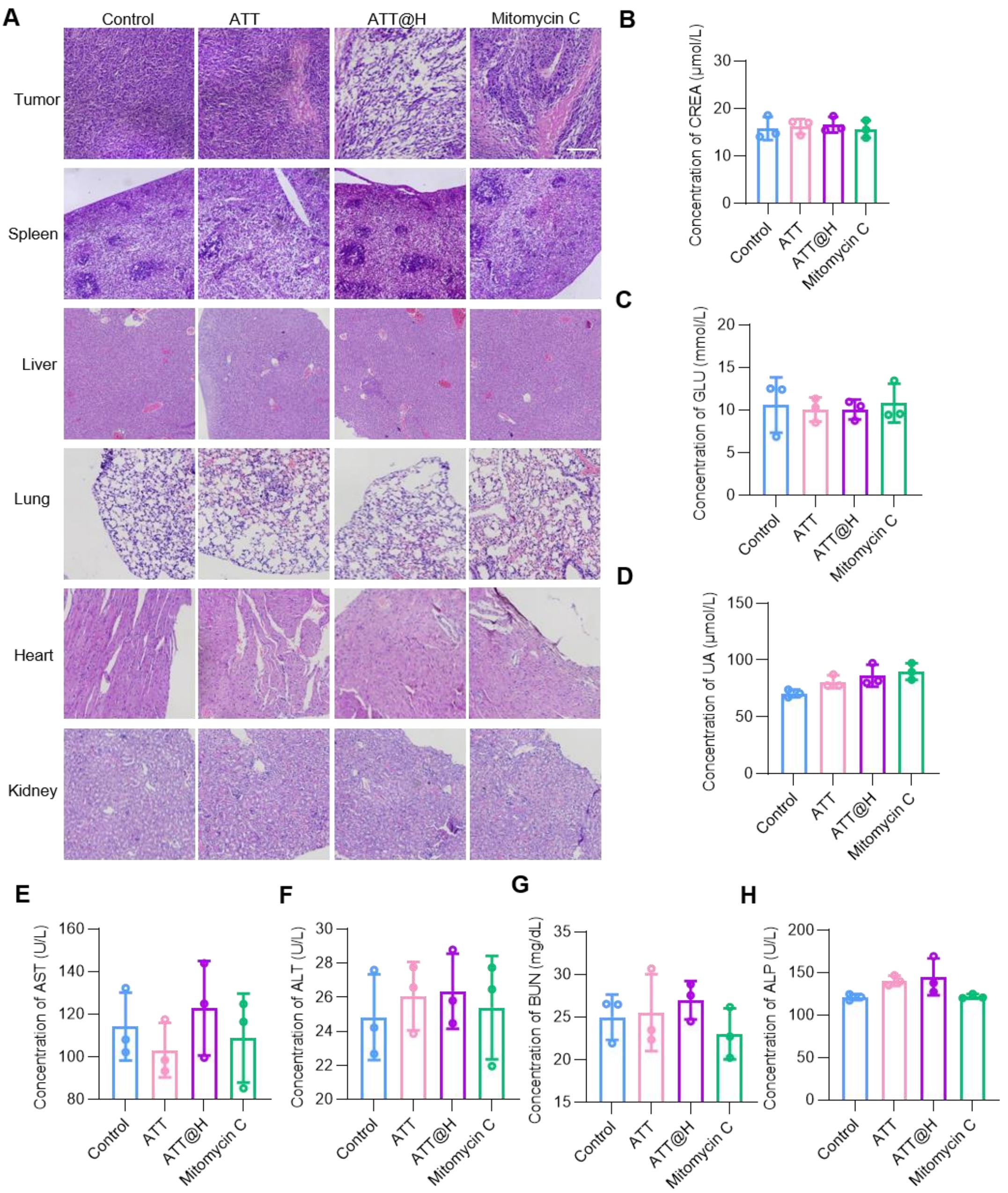

Figure S22. A) H&E staining in tumor tissues and organs from different treatments after administration for 6-week (Scale bars: 50 μm). B-H) Blood test results of different groups (n = 3). CREA, creatinine; GLU, glucose; BUN, blood urea nitrogen; AST, aspartate transaminase; ALP, alkaline phosphatase; ALT, alanine transaminase; UA, uric acid.
